# The Molecular Basis of Freshwater Adaptation in Prawns: Insights from Comparative Transcriptomics of Three *Macrobrachium* Species

**DOI:** 10.1093/gbe/evz045

**Published:** 2019-03-06

**Authors:** Md Lifat Rahi, Peter B Mather, Tariq Ezaz, David A Hurwood

**Affiliations:** 1 Science and Engineering Faculty, School of Earth Environment and Biological Sciences (EEBS), Queensland University of Technology (QUT), Brisbane, Queensland, Australia; 2 Wildlife Genetics Laboratory, Institute for Applied Ecology, University of Canberra, Australian Capital Territory, Australia

**Keywords:** RNA-Seq, adaptation genomics, differential gene expression, ALD, ELD

## Abstract

Elucidating the molecular basis of adaptation to different environmental conditions is important because adaptive ability of a species can shape its distribution, influence speciation, and also drive a variety of evolutionary processes. For crustaceans, colonization of freshwater habitats has significantly impacted diversity, but the molecular basis of this process is poorly understood. In the current study, we examined three prawn species from the genus *Macrobrachium* (*M. australiense*, *M*. *tolmerum*, and *M*. *novaehollandiae*) to better understand the molecular basis of freshwater adaptation using a comparative transcriptomics approach. Each of these species naturally inhabit environments with different salinity levels; here, we exposed them to the same experimental salinity conditions (0‰ and 15‰), to compare expression patterns of candidate genes that previously have been shown to influence phenotypic traits associated with freshwater adaptation (e.g., genes associated with osmoregulation). Differential gene expression analysis revealed 876, 861, and 925 differentially expressed transcripts under the two salinities for *M*. *australiense*, *M*. *tolmerum*, and *M*. *novaehollandiae*, respectively. Of these, 16 were found to be unannotated novel transcripts and may be taxonomically restricted or orphan genes. Functional enrichment and molecular pathway mapping revealed 13 functionally enriched categories and 11 enriched molecular pathways that were common to the three *Macrobrachium* species. Pattern of selection analysis revealed 26 genes with signatures of positive selection among pairwise species comparisons. Overall, our results indicate that the same key genes and similar molecular pathways are likely to be involved with freshwater adaptation widely across this decapod group; with nonoverlapping sets of genes showing differential expression (mainly osmoregulatory genes) and signatures of positive selection (genes involved with different life history traits).

## Introduction

Understanding interactions between living organisms and their surrounding environments is central to answering many ecological and evolutionary questions but it remains a formidable task to fully uncover the genetic and/or genomic basis of adaptive processes (Alvarez et al. 2005). It is widely accepted that life evolved first in the sea, following which over various evolutionary time scales, different groups then invaded and colonized freshwater (Clark 2006). The transition from marine to freshwater is considered to be an important step that has impacted the diversity of life now present on earth (Betancur-R et al. 2012; Vogt 2013; Rahi et al. 2017). In a newly invaded habitat, organisms typically deploy an array of adaptive responses to counteract any adverse impacts from environmental stressors (Yampolsky et al. 2014). Both phenotypic plasticity and genetic changes, in a complementary manner or independently, can contribute to this process (Jones et al. 2012; Kozak et al. 2014). Previous investigations have suggested that initial responses result from behavioral and/or phenotypic plasticity which is then followed by genetic assimilation (Wray 2013; Feldman et al. 2016; Zhu et al. 2016; Rahi et al. 2018).

Adaptation at the molecular level can occur via two major mechanisms: initially by highly plastic regulatory changes in key genes which are shaped by environmental factors (Stapley et al. 2010; Wray 2013; Berens et al. 2015); and/or via fixation of adaptive mutations over prolonged evolutionary time scales (Orr 2005; Rogl et al. 2018). Emergence of novel genes (“taxonomically restricted” or “orphan” genes) is also considered to be an important adaptive mechanism and can play a key role in lineage-specific adaptation to novel environments (Tautz and Domazet-Lošo 2011; Palmieri et al. 2014). In theory, orphan genes can emerge over relatively short evolutionary time scales, quickly become functional, and can provide the raw material for selection to act on, leading to adaptation (i.e., through neofunctionalization) (Colbourne et al. 2007; McLysaght and Hurst 2016).

To date, only 15 out of 26 extant multicellular animal phyla that first evolved in the sea have successfully adapted to inland freshwaters (Vogt 2013). In particular, a number of crustacean groups are well known for their relative success in repeated colonization of continental freshwaters from a marine ancestral state (Alvarez et al. 2005; Freire et al. 2008; Rahi 2017). Crustacean taxa exhibit ample habitat diversity and representatives are found in a diverse array of aquatic habitats from fresh to marine waters (Vogt 2013). In certain crustacean groups, freshwater adaptation is considered to be still ongoing because many of these freshwater taxa with marine ancestry still require brackish or marine water to complete their life cycles, mainly for larval development (Betancur-R et al. 2012). Major traits that evolved in freshwater crustaceans include diverse osmoregulatory patterns, large egg size, reduced fecundity, and abbreviation in number of larval developmental stages (Wowor et al. 2009; Furriel et al. 2010; Vogt 2013). The osmoregulatory processes allow regulation of internal ionic conditions, whereas the later processes facilitate reproduction via larval transport.

Although osmoregulation is considered to be the principal mechanism for adaptation to different osmotic conditions (Lee and Bell 1999; DeFaveri et al. 2011; Kozak et al. 2014; Velotta et al. 2017), it is not however, the only mechanism for freshwater adaptation; other mechanisms can also play important roles. The molecular mechanisms and pathways involved with freshwater adaptation have not been investigated thoroughly in crustaceans, although a number of studies on fish have concluded that changes to expression of osmoregulatory genes play the vital roles (DeFaveri et al. 2011; DeFaveri and Merila 2014; Brennan et al. 2015; Velotta et al. 2015, 2017; Moshtaghi et al. 2018). Investigation of molecular pathways can also provide important insights for understanding the crustacean freshwater adaptation processes because the actions of specific genes are implemented via these pathways (Berens et al. 2015). Adaptive mechanisms either can be conserved across different lineages or may be divergent depending on the phenotype as well as the species (Kozak et al. 2014; Berens et al. 2015; Rahi et al. 2018). Whether the same or different molecular pathways/mechanisms have driven for adaptation to variable osmotic environments in aquatic crustaceans remains unknown.

Freshwater prawns in the genus *Macrobrachium* constitute one of the most diversified and speciose crustacean groups, with 258 extant species currently described globally (Vogt 2013). The majority of extant *Macrobrachium* taxa occur in freshwater during the adult stage but require brackish or sea water to complete larval development (Murphy and Austin 2005; Pileggi and Mantelatto 2010). Phylogenetic studies of this genus have reported multiple clades suggesting at least nine independent invasions into freshwater from marine ancestors worldwide (Murphy and Austin 2005; Wowor et al. 2009; Pileggi and Mantelatto 2010). Currently, only 25 species are known to be able to complete their entire life cycle in freshwater, and only a few species are known to inhabit brackish and/or sea water for their whole life cycle (Wowor et al. 2009; McNamara et al. 2015). Depending on life history traits, *Macrobrachium* species are categorized into two groups: abbreviated larval development (ALD) type and extended larval development (ELD) type (Short 2004). ALD species can complete entire life cycle in freshwater, are characterized by having only a few (20–250) large eggs, and have only 1–3 larval developmental stages (Rahi 2017). ELD species in contrast require brackish or sea water to complete larval development, have numerous smaller eggs (500–10,000), and many (8–15) larval developmental stages (Murphy and Austin 2005; Wowor et al. 2009; Moshtaghi et al. 2016). It is also widely accepted that ALD species are most likely to be the oldest invaders of freshwater because there has been sufficient time for their freshwater adaptation to be complete (Vogt 2013; Rahi et al. 2017). As such, freshwater adaptation in ELD species can be considered an ongoing process as they still require brackish/marine water to complete larval development (Pileggi and Mantelatto 2010). Because the genus *Macrobrachium* is monophyletic but with many clades identified within the phylogeny containing both ALD and ELD species (Wowor et al. 2009; Vogt 2013) reflecting multiple independent freshwater invasions (Murphy and Austin 2005), this group provides an ideal opportunity for exploring the transition from marine to freshwater environments. All of the globally distributed ALD species generally display the same suite of phenotypic life history traits (Moshtaghi et al. 2017), indicating that these traits are likely to be required for, or the result of, complete adaptation to freshwater. An investigation including both ALD and ELD *Macrobrachium* species under the same experimental setting can contribute to deciphering a more comprehensive understanding of the molecular evolution of adaptation to freshwater by crustaceans more widely. In this regard, a comparative transcriptomics study can be preferential to genomics-based methods in *Macrobrachium* taxa because this group possesses very large genomes (2–4 times larger than human genome) (Baldo et al. 2011; Berdan et al. 2015; Gregory 2016).

Here, we compared the transcriptomes from three *Macrobrachium* species (*M*. *australiense*, *M. tolmerum*, and *M*. *novaehollandiae*) that represent various levels of freshwater adaptation to different osmotic ranges. Our aim was to 1) identify potential candidate genes (including lineage-specific novel or orphan genes) that may influence freshwater adaptation, 2) evaluate differential expression of target genes, 3) characterize molecular pathways involved, and 4) test for signatures of selection on identified candidate genes.

## Materials and Methods

### Species Collection and Transportation

Adult individuals from three *Macrobrachium* species representing different positions across the ELD to ALD spectrum were collected from their respective natural environments. For each species, 24–30 individuals were collected using baited traps. Collection sites and details of each species are presented in table 1. Live prawns were transferred to plastic containers supplied with aeration immediately after capture and then brought to the Marine Laboratory at the Earth, Environment and Biological Sciences (EEBS) School at Queensland University of Technology (QUT), Brisbane. Prawns were maintained in aerated tanks in water collected from each sampling site (respective salinities are indicated in table 1) until required for further analysis. Species examined belong to three different clades in the *Macrobrachium* phylogeny (Murphy and Austin 2005; Wowor et al. 2009) and thus, likely represent independent evolutionary invasions of freshwater within the genus. Thus, the three species provide ideal candidates for exploring the molecular mechanisms that are involved with an adaptive response to variable environmental salinity conditions.

**Table 1:** Life History Details of the Three *Macrobrachium* Species Compared in the Current Study

Traits	Species		
	*Macrobrachium australiense*	*Macrobrachium tolmerum*	*Macrobrachium novaehollandiae*
Adult habitat	Freshwater (0‰)	Coastal freshwater creeks (0‰)	Brackish to sea water (10–30‰)
Larval habitat	Same as adult	Brackish to sea water (15–30‰)	Sea water (30–35‰)
Developmental stages	2–3 larval stages	12–14 larval stages	14–15 larval stages
Egg size (fecundity)	Large 1.7–2.2 mm (80–200)	Small 0.8–1.0 mm (800–5,000)	Small 0.6–0.9 mm (1,000–4,000)
Larval duration from hatching to PL stage (days)	3–5	60–90	75–100
Life history category	Abbreviated (ALD) type	Extended (ELD) type	Extended (ELD) type
Distribution	Across mainland Australia	Coastal drainages of Queensland	Coastal brackish-water creeks in Queensland
Sampling location	Stony Creek (26°88′98″S, 152°73′22″E)	Cooroonpah Creek (27°46′90″S, 153°44′38″E)	Oxley Creek (27°54′61″S, 152°99′48″E)
Salinity level at point of sample collection (‰)	0	0	15
Temperature during sample collection (°C)	22	23	23

### Experimental Tank Preparation and Acclimation

Six glass tanks (40 l) were used for trials of each species. Small plastic tubes (≈15 cm in length), sand and small rocks were placed in each tank to create artificial habitat. Prawns use plastic tubes as shelter to avoid predation by other prawns during moulting. Tanks were maintained with continuous aeration across the experimental period and biofilters were also placed in each tank to maintain optimum water quality. In each experimental tank, 4–5 prawns were allocated randomly and maintained for a 14 day acclimation period. Salinity levels were then raised 3‰ per day for *M*. *australiense* and *M*. *tolmerum*, whereas salinity level was decreased by 3‰ per day for *M*. *novaehollandiae*. This process continued until each species inhabited tanks with two different experimental salinity levels (0‰ and 15‰). For *M*. *australiense* and *M*. *tolmerum*, 0‰ was the control condition, whereas 15‰ was the control for *M*. *novaehollandiae*. To increase salinity level in each experimental tank, saline water (60‰) was prepared by mixing commercially available sea salt (Tropic Marine Pro-Reef) with dechlorinated tap water. To reduce salinity level to 0‰ for *M*. *novaehollandiae*, dechlorinated tap water was slowly added in the tanks. After achieving target experimental salinity levels, prawns were maintained at the two salinity levels for 6 weeks. Prawns were fed with frozen brine shrimp (*Artemia*) once daily in the late afternoon. We considered that this experimental set should be sufficient to precisely examine chronic salinity induced differential expression pattern of the candidate genes, whereas acute changes due to specific direction of salinity transfer would largely be ignored.

### RNA Extraction, cDNA Library Preparation, and Illumina Sequencing

Prawns were euthanized on ice and then dissected to obtain fresh tissues. In order to obtain sufficient tissue and to get wide representation of expressed genes, gill, antennal gland, eye stalk, hepatopancreas, and intestinal muscle tissues were dissected and pooled together from each individual. We applied this pooling strategy to all three species to minimize interspecies gene expression biases. It was also considered that if there is any potential bias in gene expression associated with pooling multiple tissues, it will likely impact all three species equally. Pooled tissues from each individual were crushed with liquid nitrogen to make a fine powder. Total RNA was extracted from powdered tissues using a TRIzol/chloroform extraction method (Chomczynski and Mackey 1995) followed by RNA purification using an ISOLATE II RNA Mini Kit (Cat # 52072, Bioline, UK). The RNA kit protocol also included a column DNA digestion step to remove genomic DNA contamination. For RNA extraction, 10–12 individuals from the two salinity conditions per species were used. Total RNA quality was checked using 2% agarose gel electrophoresis, NanoDrop 2000 Spectrophotometer (Thermo Scientific) and Bioanalyzer (Agilent 2100, version 6). The best quality (top three samples from each salinity for each species) RNA samples were used in all subsequent steps (table 2).

**Table 2:** Experimental Design for Next Generation Sequencing (NGS) Analysis

	*Macrobrachium australiense*		*Macrobrachium tolmerum*		*Macrobrachium novaehollandiae*	
	**0**‰	**15**‰	**0**‰	**15**‰	**0**‰	**15**‰
Body weight of the prawns (g)	9–11	9–12	8–10	8–11	12–15	11–14
No. of individuals used for RNA extraction	12	10	11	12	10	12
No. of individuals used for cDNA library preparation	3	3	3	3	3	3
No. of cDNA libraries sequenced in Illumina	3	3	3	3	3	3

For transcriptome sequencing, cDNA libraries were prepared following TrueSeqV1 Standard mRNA Sample Prep kit (Illumina, USA) instructions using 4 µg of total RNA. cDNA libraries were prepared according to the methods outlined in an earlier study (Rahi et al. 2017) that included 1) poly(A) tail mRNA capture and fragmentation, 2) cDNA synthesis from size selected fragments, 3) subsequent purification of cDNA fragments, 4) repair and adenylation of 3′ ends, 5) bar coding of each cDNA library, and 6) enrichment of cDNA libraries via 15 cycles of polymerase chain reaction (PCR) amplification. The quality of each cDNA library was assessed using Bioanalyzer (Agilent 2100, USA); quantity was estimated using Qubit2.0 Fluorometer (Invitrogen, Life Technologies) and RT-qPCR (BJS Biotechnologies, UK). In total, 18 cDNA libraries were prepared (table 2). Equimolar quantities of all cDNA libraries were used for sequencing on a NextSeq 500 Sequencer (Illumina, San Diego, CA).

### De Novo Transcriptome Assembly, Blast Analysis, and Annotation

Overall quality of Illumina raw sequence reads was checked using FastQC software (Andrews 2010). Trimmomatic software (Bolger et al. 2014) was used to remove extraneous (adapter) sequences and for quality filtering of raw sequence reads applying default settings prior to de novo assembly. Clean reads from each cDNA library were used to perform de novo assembly using Trinity software applying default settings (Haas et al. 2013, version 2014-04-13p1) to generate longer contigs/transcripts. For each species, six cDNA libraries (three from 0‰ and three from 15‰) were pooled prior to de novo assembly to generate a reference transcriptome for each species, separately. Transcriptome assembly completeness was then assessed using CEGMA (Parra et al. 2007) and BUSCO (Simao et al. 2015) software. De novo assembled transcripts were blast searched against the NCBI NR databases using BlastX applying an *E* value threshold at 1e^−6^ to identify putative homologs and/or orthologs. Gene ontology (GO) annotations for describing cellular components, molecular function, and biological processes were obtained using BLAST2GO Pro software (Conesa et al. 2005).

### Read Mapping and Differential Expression Analysis

Paired-end raw reads were aligned to the reference transcriptome for each species separately using Bowtie (Langmead and Selzberg 2012; version 2.1.0). Raw reads were then mapped back to the transcriptomes for relative transcript abundance estimation (for read counts) using RSEM (Li and Dewey 2011). Read counts that mapped to assembled contigs were normalized subsequently to fragments per kilobase per million (FPKM) values using the TMM read normalization method. FPKM parameter values for a subset of genes were visualized with box and whisker plots using statistical package R (version 3.1.2) to compare expression patterns at different salinities among species. Raw read counts were then analyzed with EdgeR (Robinson et al. 2010) from Trinity and Bioconductor repositories (Gentleman et al. 2004) to test for transcript differential expression patterns (at FDR ≤ 0.001) comparing between salinities for each species (Haas et al. 2013; Berens et al. 2015). Principal component analysis (PCA) was then performed for each species including all differentially expressed transcripts to visualize the effects of salinity on expression patterns (Linde et al. 2015). A single PCA was also performed for the 783 transcripts commonly expressed among the three species. In addition, a PCA was also conducted using logarithmic FPKM values from selected candidate genes and novel transcripts to assess species × salinity interactions. For each species, all differentially expressed transcripts were extracted from the data and blasted against each other to identify common transcripts between species and species-specific transcripts/genes. As the Trinity assembler produces many isoforms of the same genes, we loaded all of the differentially expressed transcripts in the UCLUST program (Edgar 2010) to eliminate redundant sequences, revealing the actual number of differentially expressed genes.

### Functional Enrichment and Molecular Pathway Analyses

We used Trinotate (Finn et al. 2011; Brekhman et al. 2015) and GOSeq (Young et al. 2010) software from the Trinity repository for functional enrichment analysis of each species. Functional enrichment analysis was performed on the differentially expressed transcripts from each species to determine enrichment (*P* value ≤ 0.01) of specific GO term categories and metabolic pathways. Kyoto Encyclopedia of Genes and Genomes (KEGG) for each species were obtained using BLAST2GO Pro software (Conesa et al. 2005). KOBAS software (Xie et al. 2011) was then used to identify and annotate enriched molecular (KEGG) pathways for each species.

### Identification of Novel Transcripts

We used all of the differentially expressed transcripts from each species for this analysis. To identify the orphan transcripts/genes, we used a relaxed cutoff parameter (stringency of *E* value 1e^−3^) for blasting (Tautz and Domazet-Lošo 2011). We then extracted differentially expressed transcripts for each species that had not received blast hits. In total, 16 differentially expressed novel transcripts were identified that were common to all three *Macrobrachium* species examined here. Potential novel genes were further checked in open reading frame Finder to identify protein coding regions in order to confirm that these transcripts were functional. We aligned the coding sequences of novel transcripts identified in the *Macrobrachium* species studied here to check that all species possessed the same coding lengths. Unknown genes (differentially expressed transcripts without blast hits) were further blasted against the *Daphnia* genome to confirm that they were not present in the closest relative with an annotated genome.

### Genes Identified A Priori

Prior to the study, we prepared a list of 43 candidate genes from an in depth literature review that were known to be involved with freshwater adaptation in a variety of aquatic crustacean species (supplementary table S1, Supplementary Material online). Genes that were differentially expressed and positively selected from the list (supplementary table S1, Supplementary Material online) were considered to be the most important because they were known to play significant functional roles in freshwater adaptation in a wide range of crustacean species. We extracted each of the differentially expressed genes from this list (supplementary table S1, Supplementary Material online) for each target species: *M*. *australiense*, *M. novaehollandiae*, and *M*. *tolmerum*. FPKM values for differentially expressed genes were then converted to logarithmic fold change to better assess their patterns. Novel transcripts were also treated the same way.

### Signatures of Selection on Protein Coding Regions

Sequences of all the differentially expressed genes (205) (including the 16 orphan genes) and 43 a priori genes were also obtained for this analysis from another ALD species (*M*. *koombooloomba*) examined in an earlier study (Rahi et al. 2017). We applied two different approaches to investigate signatures of selection acting on coding sequences of genes that were likely to be involved with freshwater adaptation in *Macrobrachium* species. We also compared the sequences of orphan genes and/or transcripts between the target species to investigate selection patterns. Initially, all potential genes from each species were aligned separately using MAFFT (Katoh and Standley 2013). We used the Yang and Nielsen (2000) counting method in the PAML V4.7 software package (Yang 2007) to estimate the *d*_N_/*d*_S_ ratio that is the number of nonsynonymous substitutions per nonsynonymous site (*d*_N_) to number of synonymous substitutions per synonymous site (*d*_S_) and whether this ratio deviates from neutral expectation. A *d*_N_/*d*_S_ ratio of <1 indicates purifying selection, =1 indicates neutral evolution, whereas >1 indicates positive selection (Dunning et al. 2016). For this method, *d*_N_/*d*_S_ is estimated from pairwise comparisons between coding sequences, a process that provides a single estimate for the entire open reading frame, whereas considering only fixed differences between species without the need for a comparison with a closely related outgroup. *P* values for detecting significant deviations from 1 were adjusted for multiple comparisons using Bonferroni correction method (Rice 1989) based on the number of species pairwise comparisons (*α* = 0.05/6 = 0.008).

Additionally, the fraction of nonsynonymous substitutions (*f*_N_) was calculated using the equation, *f*_N_ = *d*_N_/(*d*_N_ + *d*_S_) according to Xie et al. (2011). We calculated *f*_N_ because *d*_N_/*d*_S_ values can be extremely large or may be uninformative if *d*_S_ is, or close to, zero. In this regard, calculating *f*_N_ can compensate for this problem and can improve analysis by estimating the rate of mutation as opposed to using the absolute number of mutations (Yang and Nielsen 2000).

### Identification of Candidate Genes Involved with Freshwater Adaptation

We focused on GO terms (osmoregulation, ion exchange, cell volume, body fluid, water channel, stress response, egg size, larval development, etc.), differential gene expression patterns, functional enrichment analysis, signatures of positive selection, and a literature search to identify candidate genes. All the differentially expressed genes/transcripts including the novel transcripts, and positively selected genes were considered to be the key candidates involved with freshwater adaptation in *Macrobrachium* lineages.

### RT-qPCR Assay for the Validation of Differential Gene Expression

For this validation study, we used the same RNA samples that were considered for cDNA library preparation. In total, ten individuals were sampled randomly from each salinity for each species. Total RNA was converted to cDNA using a SensiFAST cDNA synthesis kit (Cat # BIO-65054, Bioline, UK). Specific primers were designed for a reference gene (18S) and eight different genes including two orphan transcripts (supplementary table S2, Supplementary Material online) using Primer3 software (Untergasser et al. 2012). Prior to primer design, we aligned protein coding sequences from each species using MAFFT (Katoh and Standley 2013) and then primers were designed from highly conserved regions to ensure that the same set of primers amplify in all three species. RT-qPCR reactions were then performed using SensiFAST SYBR No-ROX Mix (Bioline, UK) in a thermal cycler R-Corbett (RG-6000, Australia). PCR conditions were maintained at: 95 °C for 2 min for polymerase activation and 40 cycles (denaturation at 95 °C for 5 s, annealing at 58–62 °C for 20 s and an extension step at 72 °C for 20 s). Relative gene expression values were then obtained using a delta–delta method following standard protocols (Pfaffl 2001; Velotta et al. 2015). We used 18S as a reference because expression of this gene did not change between salinities for each species. Moreover, 18S has been found to be a suitable reference gene for crustaceans in a number of earlier studies (Faleiros et al. 2010; Havird et al. 2014; Moshtaghi et al. 2018; Rahi et al. 2017; Aziz et al. 2018). Relative gene expression values were analyzed in SPSS (version 22) at 5% level of significance.

## Results

### Nextgen Sequencing, De Novo Transcriptome Assembly, and Annotations

High throughput Illumina sequencing yielded approximately 300 million, 250 million, and 245 million copies of 75 base paired-end sequences for *M*. *australiense*, *M*. *tolmerum*, and *M*. *novaehollandiae*, respectively (table 3). The number of de novo assembled contigs, assembly completeness, and additional annotation statistics for each target species are presented in table 3. An assay of transcriptome assembly revealed that completeness ranged from 97% to 98%, indicating a high quality de novo assembly for each species. The top hit species distribution chart (supplementary fig. S1, Supplementary Material online) showed that all three *Macrobrachium* species received the highest blast matches with *Daphnia*. Although *Daphnia* is phylogenetically very distantly related to *Macrobrachium*, to date, this is the only crustacean species with a high quality complete genome assembly available. The three *Macrobrachium* species screened here, all showed similar patterns of top most abundant GO term categories (supplementary fig. S2, Supplementary Material online); for example abundance of “ion binding” was 11,165, 10,508, and 10,988 in the *M*. *australiense*, *M*. *tolmerum*, and *M*. *novaehollandiae* annotated transcriptome data sets, respectively.

**Table 3:** Illumina Sequencing, De Novo Assembly, and Annotation Statistics

Parameters	Species		
	*Macrobrachium australiense*	*Macrobrachium tolmerum*	*Macrobrachium novaehollandiae*
Illumina raw reads	298,951,912	247,389,884	243,465,076
No. of reads after trimming	273,911,694	238,564,705	236,785,876
Assembled contigs	123,396	134,227	131,647
CEGMA completeness (%)	97.38	96.9	97.8
BUSCO completeness (%)	98.1	97.8	97.9
N50 value	2,182	2,064	2,013
Mean contig length	978	930	811
Median contig length	400	396	380
Range of contig length	201–28,827	201–27,323	201–20,399
Contigs with blast hits	38,597	37,721	41,655
Contigs annotated	24,722	24,673	25,553

### Differential Expression Patterns

**Fig. 1.**
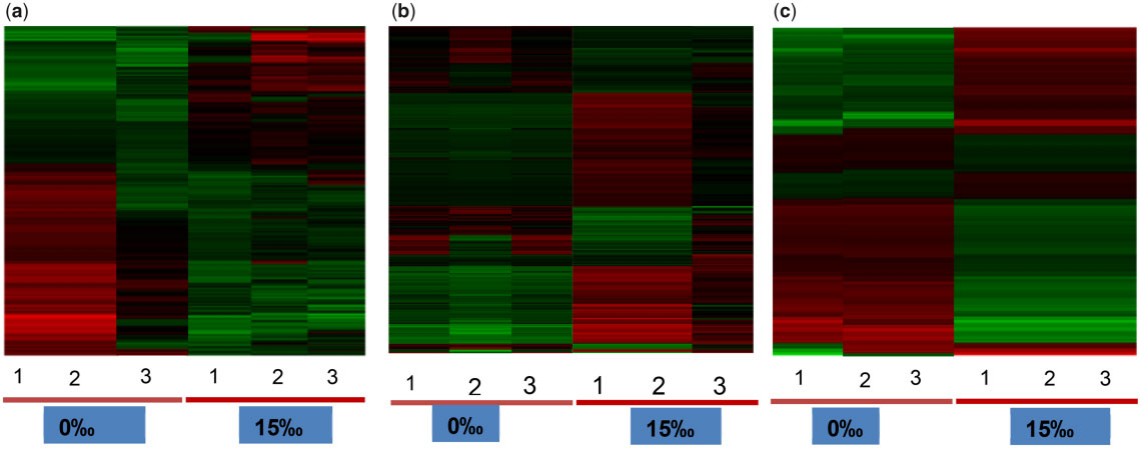
—Heatmap showing differential expression pattern of transcripts at 0‰ and 15‰ salinities for three different *Macrobrachium* species. (*a*) *M*. *australiense* (876 transcripts), (*b*) *M*. *tolmerum* (861 transcripts), and (*c*) *M*. *novaehollandiae* (925 transcripts).

**Fig. 2.**
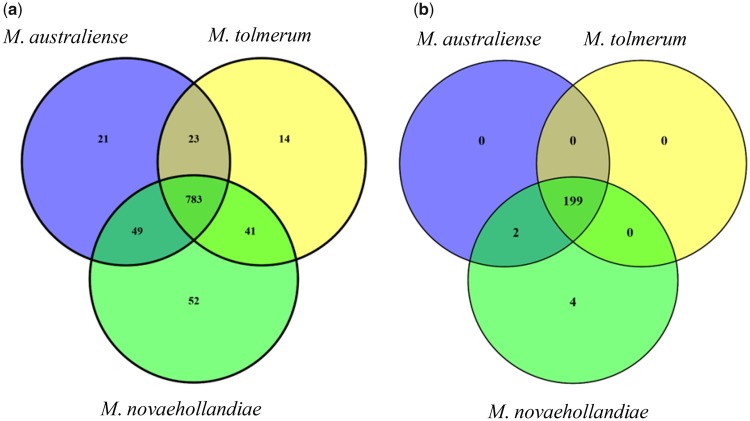
—Venn diagram showing the number of common and species-specific differentially expressed: (*a*) transcripts and (*b*) genes. Eight hundred seventy-six transcripts are different isoforms or subunits from 201 genes in *Macrobrachium australiense*, 861 transcripts from 199 genes in *Macrobrachium tolmerum*, and 925 transcripts from 205 genes in *Macrobrachium novaehollandiae*.

**Fig. 3.**
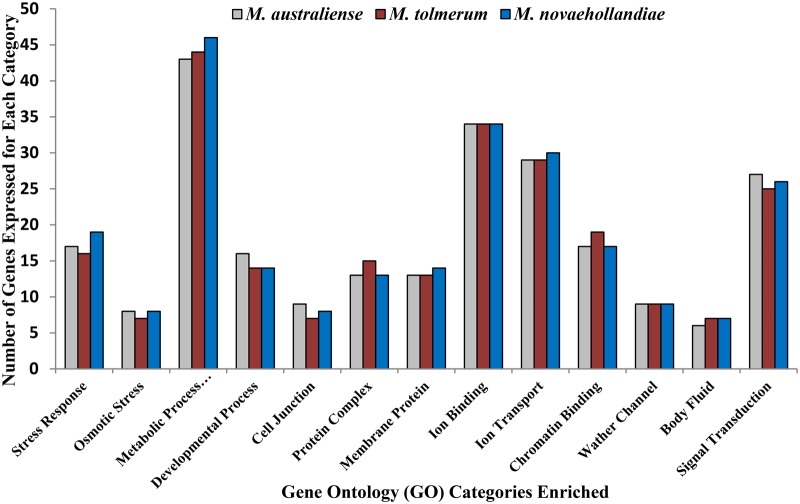
—Number of differentially expressed genes under the functionally enriched (at *P* value <0.01) GO term categories.

**Fig. 4.**
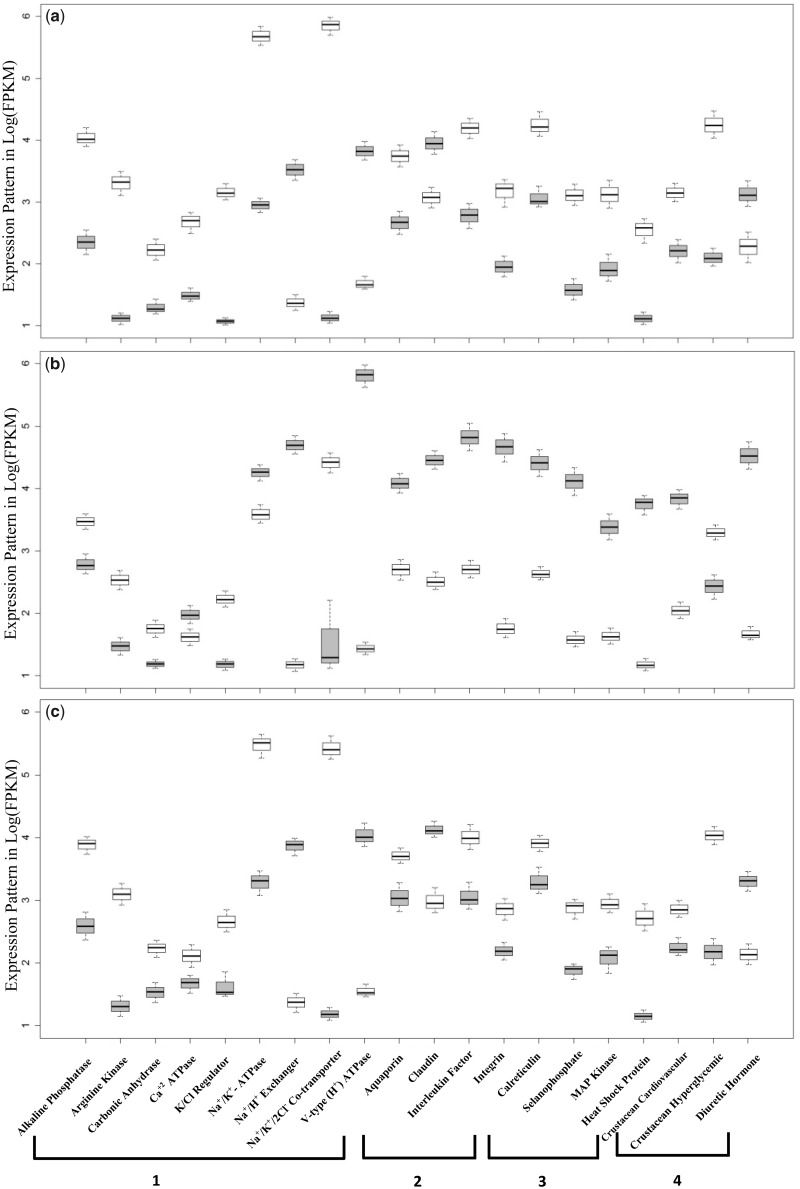
—Box and Whisker plots showing differential expression pattern (in log[FPKM]) of some (top 20) preidentified genes at 0‰ (gray boxes) and 15‰ (white boxes): (*a*) *Macrobrachium australiense*, (*b*) *Macrobrachium novaehollandiae*, and (*c*) *Macrobrachium tolmerum*. Candidate genes on the *x* axis are grouped by functional role as 1_** **_=_** **_osmoregulatory genes; 2_** **_=_** **_cell volume and cellular junction maintaining genes; 3_** **_=_** **_stress response genes; and 4_** **_=_** **_body fluid (hemolymph) regulating genes.

In total, 876, 861, and 925 transcripts were differentially expressed for *M*. *australiense*, *M*. *tolmerum*, and *M*. *novaehollandiae*, respectively (figs. 1 and 2). In total, 783 transcripts were found to be differentially expressed in all three species, whereas 14, 21, and 52 transcripts showed species-specific differential gene expression patterns, respectively (fig. 2). Most of the differentially expressed transcripts were found to be different copies or subunits of a relatively smaller number (199–205) of genes (the actual number of genes expressed is shown in figs. 2 and 3). Higher number of commonly expressed transcripts compared with a lower number of species-specific transcripts is consistent in general, with a conserved response to variable salinity change across the genus. Out of 43 preidentified genes, 35 were differentially expressed, whereas 17 were positively selected (table 4 and supplementary tables S4 and S5, Supplementary Material online); 9 genes showed both differential expression and positive selection patterns indicating that the same genes are involved with freshwater adaptation across the crustacean lineages. Figure 3 shows the logarithmic fold changes in expression values (FPKM values) for the 20 most differentially expressed preidentified genes (from supplementary table S1, Supplementary Material online) for each species under the two experimental salinity conditions. Differentially expressed genes in the three *Macrobrachium* species (0‰ vs. 15‰ conditions) were found to be involved with osmoregulation, body fluid and water channel regulation, control of cell volume regulation and cellular junction, stress response, metabolic and signaling processes, energy budgeting (figs. 3 and 4 and supplementary tables S1, S3, and S4, Supplementary Material online), etc. RT-qPCR assay of eight genes (including two orphan genes/transcripts) confirmed the validity of respective gene expression patterns (supplementary fig. S7, Supplementary Material online) as we observed significant differences (*P* < 0.05) in expression for these genes under the two experimental salinities in each species.

**Table 4:** Enriched Molecular Pathways and Number of Differentially Expressed Transcripts

Pathway ID	Pathway Description	No. of Annotated Sequences		
		*Macrobrachium australiense*	*Macrobrachium tolmerum*	*Macrobrachium novaehollandiae*
Path:ko00061	Fatty acid biosynthesis	236	230	239
Path:ko01040	Biosynthesis of unsaturated fatty acid	103	109	105
Path:ko00062	Fatty acid elongation	91	90	91
Path:ko00533	Glycosaminoglycan biosynthesis	172	170	171
Path:ko00601	Glycosphingolipid biosynthesis	130	132	127
Path:ko00590	Arachidonic acid metabolism	147	144	151
Path:ko00564	Glycerophospholipid metabolism	161	164	167
Path:ko00565	Ether lipid metabolism	73	73	75
Path:ko00072	Degradation of ketone bodies	42	39	43
Path:ko04151	P13-AKT signaling pathway	198	194	203
Path:ko00230	Purine metabolism	205	206	210

Out of all the differentially expressed transcripts, 16 were found to be orphan transcripts that were highly differentially expressed between the 0‰ and 15‰ salinities in the three *Macrobrachium* species compared here (supplementary fig. S3, Supplementary Material online). These orphan transcripts or taxonomically restricted genes potentially play important adaptive roles in variable osmotic environments and ultimately may contribute to freshwater adaptation. Each of the 16 transcripts could represent discrete whole functional genes or potentially may be fragments of a smaller number of unidentified unique genes.

**Fig. 5.**
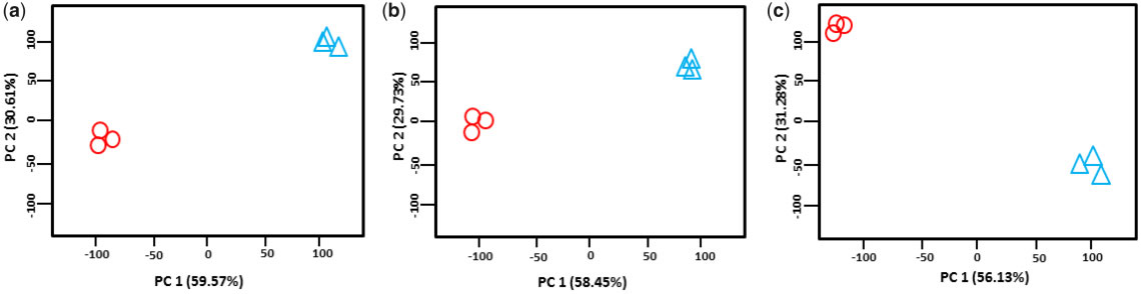
—PCA plots of all differentially expressed transcripts at 0‰ (circles) and 15‰ (triangles) for the three *Macrobrachium* species: (*a*) *M*. *australiense*, (*b*) *M*. *tolmerum*, and (*c*) *M*. *novaehollandiae*.

PCA plots of all differentially expressed transcripts (fig. 5) and common 783 transcripts shared between the three species (supplementary fig. S6, Supplementary Material online) revealed a conserved response among species, where *M*. *novaehollandiae* showed the reverse pattern (higher expressions at 0‰) compared with *M*. *australiense* and *M*. *tolmerum* (higher expression at 15‰). Of note, *M*. *tolmerum* showed higher expression levels (higher FPKM values) compared with *M*. *australiense* in freshwater and the reverse pattern was observed at 15‰. Larval development of *M*. *tolmerum* occurs in sea water and this species therefore is more tolerant of raised salinity conditions. PCA results for species and salinity interactions based on preidentified genes and on orphan transcripts also revealed that *M*. *australiense* and *M*. *tolmerum* showed similar response, whereas *M*. *novaehollandiae* showed opposite response (supplementary figs. S4 and S5, Supplementary Material online).

### Functional Enrichment Analysis

Functional enrichment analysis of the differentially expressed genes revealed that 13 different GO terms were significantly enriched (at *P* < 0.01) between the 0‰ and 15‰ conditions in all three *Macrobrachium* species (fig. 3). For all three species, the same functional GO term categories were enriched with each showing a very similar number of genes expressed under each category. In total, 201, 199, and 205 different genes were found to be expressed under 13 functionally enriched GO categories in *M*. *australiense*, *M*. *tolmerum*, and *M*. *novaehollandiae*, respectively. Given that within *Macrobrachium* the three target species are only distantly related phylogenetically (Wowor et al. 2009), these results indicate that common functional, molecular and/or biological mechanisms likely contributed to adaptive response to variable osmotic niches across the *Macrobrachium* genus more widely.

### Molecular Pathway Mapping

In total, 133, 134, and 136 molecular pathways were identified from KEGG for *M. australiense*, *M*. *tolmerum*, and *M*. *novaehollandiae*, respectively. KEGG pathway mapping of all the differentially expressed transcripts revealed that 11 different molecular pathways were enriched for the three *Macrobrachium* species studied here (table 4). Each of the enriched pathways was characterized by a number of differentially expressed transcripts (table 4).

### Signatures of Selection Acting on Coding Sequences

Estimates of synonymous versus nonsynonymous substitutions in target species revealed that different types of selection potentially were acting on different categories of genes. Table 5 and supplementary table S5, (Supplementary Material online) identify positively selected genes between different species pairs. In total, 26 out of all genes studied here (205 differentially expressed and 43 a priori genes) showed a signature of positive selection among different pairwise species comparisons (table 5 and supplementary table S5, Supplementary Material online). The majority of the differentially expressed genes including osmoregulatory genes, cell volume regulatory genes, and all of the 16 novel transcripts showed signs of purifying selection (*d*_N_/*d*_S_ < 1) in different species comparisons. Large-scale sequence variation were observed among species for genes that were involved with water channel regulation, body fluid maintenance, egg size control, osmotic stress response, and control of larval developmental stages. An absence of positive selection was observed between pairwise ALD (*M*. *koombooloomba*) versus ALD (*M*. *australiense*) and/or ELD (*M*. *tolmerum*) versus ELD (*M*. *novaehollandiae*) species comparisons for the genes studied here. Only three genes showed signatures of positive selection for the *M*. *koombooloomba* versus *M*. *australiense* comparison; *Vitellogenin* gene (control egg size), and *Merlin* and *Mastermind* genes (determine larval developmental stages). This potentially indicates that although *M. australiense* is an ALD species, it may not have yet reached the same degree of abbreviated developmental pattern as *M. koombooloomba*. Higher numbers of synonymous mutations (*d*_S_) were obtained in ALD versus ALD and ELD versus ELD species comparisons, whereas a large number of nonsynonymous mutations (*d*_N_) were observed for ALD versus ELD species comparisons. Higher number of *d*_S_ for *M*. *australiense* versus *M*. *koombooloomba* comparisons possibly indicate that both taxa are ALD type, or potentially because they are sister taxa, sharing a recent common ancestor. In parallel, a similar result was evident in *M*. *tolmerum* versus *M*. *novaehollandiae* comparisons. Higher *d*_S_ rates for ALD versus ALD and ELD versus ELD species can also indicate exposure to similar environmental conditions over an extended evolutionary timeframe.

**Table 5:** List of the Genes Showing Effects of Positive Selection Based on Pairwise *d*_N_/*d*_S_ ratios > 1 (Only *P* Values Significant after Bonferroni Corrections Are Given)

Species Pair	Gene (Functional Roles)	*d*_N_	*d*_S_	*d*_N_/*d*_S_	*f*_N_	*P* Value
MK vs. MA (ALD vs. ALD)	Merlin (larval development)	0.0147	0.0131	1.1221	0.5288	0.004
	Mastermind (larval development)	0.0132	0.0108	1.2223	0.5520	0.006
	Vitellogenin (controlling egg size)	0.0171	0.0156	1.0962	0.5229	0.003
MK vs. MT (ALD vs. ELD)	Merlin	0.0673	0.0337	1.9970	0.6663	0.000
	Mastermind	0.0734	0.0402	1.8259	0.6461	0.000
	Midline (multiple larval developmental role)	0.0231	0.0187	1.2353	0.5526	0.002
	Selanophosphate (osmotic stress tolerance)	0.0125	0.0121	1.0331	0.5081	0.001
	Calreticulin (osmotic stress response)	0.0213	0.0204	1.0441	0.5108	0.001
	P38 MAP kinase (osmotic signal transduction)	0.0126	0.0117	1.0770	0.5185	0.003
	Interleukin (signaling for osmotic stress)	0.0215	0.0179	1.2011	0.5457	0.000
	Claudin (maintain cellular junction)	0.0368	0.0254	1.4488	0.5916	0.000
	Integrin (maintain cell volume and junction)	0.0412	0.0307	1.3421	0.5731	0.002
	Aquaporin (water channel regulation)	0.0539	0.0394	1.3680	0.5777	0.000
	Diuretic hormone (water balance)	0.0648	0.0491	1.3198	0.5689	0.001
	Hyperglycemic hormone (regulate body fluid)	0.0736	0.0486	1.5144	0.6023	0.000
	Vitellogenin	0.0829	0.0488	1.6988	0.6295	0.000
	Vitelline (controlling egg size)	0.0417	0.0402	1.0373	0.5092	0.007
	Serpin (controlling egg size and development)	0.0214	0.0195	1.0974	0.5232	0.004
	Cullin (controlling egg size)	0.0184	0.0139	1.3237	0.5697	0.001
	Plekstrin (controlling egg size)	0.0208	0.0168	1.2381	0.5532	0.002
	Growth arrest–specific protein (growth inhibition)	0.0258	0.0179	1.4413	0.6012	0.000
	Heparan sulfate 6 (growth)	0.0273	0.0207	1.3188	0.5687	0.001
	Alpha amylase (growth)	0.0698	0.0401	1.7406	0.6711	0.000
	Pantothanate flavoprotein (metabolic activity)	0.0661	0.0329	2.0093	0.7102	0.002
	Glutatheone synthetase (feeding behavior)	0.0275	0.0167	1.6467	0.6457	0.003
	RAP guanine factor 4 (energy budgeting)	0.0409	0.0291	1.4055	0.5251	0.001
	Syndecan isoform 2 (energy homeostasis)	0.0124	0.0112	1.1072	0.5123	0.006
	TKT protein (energy production)	0.0217	0.0181	1.1989	0.5201	0.002
	Ubiquitin C (metabolic process)	0.0366	0.0291	1.2577	0.5237	0.001
MK vs. MN (ALD vs. ELD)	Selanophosphate	0.0284	0.0141	2.0142	0.6682	0.000
	Calreticulin	0.0294	0.0207	1.4202	0.5868	0.001
	P38 MAP kinase	0.0186	0.0119	1.5630	0.6098	0.000
	Interleukin	0.0274	0.0185	1.4811	0.5969	0.001
	Claudin	0.0421	0.0263	1.6008	0.6155	0.001
	Integrin	0.0497	0.0307	1.6189	0.6182	0.003
	Aquaporin	0.0631	0.0395	1.5975	0.6150	0.000
	Diuretic hormone	0.0732	0.0492	1.4878	0.5980	0.000
	Hyperglycemic hormone	0.0684	0.0345	1.9826	0.6647	0.002
	Vitelline	0.0524	0.0386	1.3575	0.5758	0.000
	Vitellogenin	0.0874	0.0451	1.9379	0.6596	0.001
	Serpin	0.0253	0.0197	1.2843	0.5622	0.000
	Cullin	0.0216	0.0141	1.5319	0.6050	0.000
	Plekstrin	0.0319	0.0171	1.8655	0.6510	0.007
	Midline	0.0286	0.0189	1.5132	0.6021	0.004
	Merlin	0.0716	0.0329	2.1763	0.6852	0.001
	Mastermind	0.0772	0.0381	2.0263	0.6695	0.002
	Growth arrest–specific protein	0.0214	0.0176	1.2159	0.5602	0.000
	Heparan sulfate 6	0.0263	0.0204	1.2892	0.5217	0.004
	Alpha amylase	0.0713	0.0412	1.7306	0.6194	0.000
	Pantothanate flavoprotein	0.0682	0.0335	2.0358	0.7115	0.000
	Glutatheone synthetase	0.0298	0.0183	1.6284	0.6487	0.002
	RAP guanine factor 4	0.0426	0.0292	1.4692	0.5312	0.003
	Syndecan isoform 2	0.0139	0.0118	1.1781	0.5096	0.005
	TKT protein	0.0225	0.0181	1.2431	0.5148	0.001
	Ubiquitin C	0.0372	0.0294	1.2653	0.5301	0.000

Note.—MK, *M*. *koombooloomba*; MA, *M*. *australiense*; MT, *M*. *tolmerum*; MN, *M*. *novaehollandiae* (almost similar values were obtained for MA versus MT and MA versus MN comparisons, so, only MK versus MT and MK versus MN comparisons are presented here. MA versus MN and MA versus MT results are available in supplementary Table S5).

Based on results from functional enrichment, differential gene expression pattern, molecular pathway analysis, signatures of positive selection, and a literature survey, at least 199–205 different genes (or >800 transcripts) were identified that likely play important roles in freshwater adaptation in *Macrobrachium* species. Identified candidate genes are engaged in at least 13 different broad biological and/or physiological processes including: osmoregulation (ion transport and balance), body fluid (hemolymph) regulation, water channel regulation, control of cell volume and cellular junction, stress tolerance, change to egg size and number, change to the number of larval developmental stages, immune response, metabolic process, energy production and growth inhibition, among others. These are generally recognized as common response mechanisms to changed environmental conditions in any organism. Thus, all of the differentially expressed and positively selected genes in the current study are likely to be potential candidates for freshwater adaptation in *Macrobrachium* lineages.

## Discussion

### Candidate Genes and Molecular Mechanisms Facilitating Freshwater Adaptation

Our comparative transcriptomics study including both ALD and ELD *Macrobrachium* species identified at least 205 putative differentially expressed candidate genes representing at least 13 different biological and/or physiological processes (fig. 3) that have been implicated in processes relating to freshwater adaptation. Among these genes, we also identified 16 orphan transcripts/genes that were found to be common in all three species examined. The preidentified 43 genes represent only a small proportion (≈30%) of the identified genes, whereas the remaining 70% are involved with other biological processes including stress response and signaling, energetics, growth, feeding, metabolic and developmental processes, and immune response. Thus, responses from genes involved with many different biological processes are required for organismal survivability and long-term persistence. Therefore, all 205 genes (or 925 transcripts) identified here can be considered as important candidate genes for adaptation to different environmental salinities in *Macrobrachium*. Comparative analysis among the three *Macrobrachium* species revealed very similar patterns of results for functional enrichment (fig. 3) and molecular pathway enrichments (table 4). As the three species belong to different clades within the genus *Macrobrachium* (Murphy and Austin 2005), these results imply that similar molecular mechanisms are likely to be involved with freshwater adaptation and salinity tolerance across the genus and potentially even more broadly across decapod crustacean lineages. Different molecular pathways and/or mechanisms can either be conserved or be divergent for adaptive response, but this largely depends on the animal taxa and target phenotypes considered (Lam et al. 2014; Berens et al. 2015). Here, we observed that the same molecular pathways and/or mechanisms contributed to freshwater adaptation in all three *Macrobrachium* species.

### Differential Expression Pattern of Identified Candidate Genes

All three *Macrobrachium* species showed very similar trends in expression patterns between salinities (supplementary table S4, fig. 5, supplementary figs. S4–S6, Supplementary Material online). PCA results further indicate that all three *Macrobrachium* species are using the same genes and molecular mechanisms/pathways but in different ways to efficiently cope with salinity changes. *M*. *novaehollandiae* in this regard, is responding differently, likely due to being inhabitant of high saline environments compared with the other two species and/or even due to different directions of salinity transfer. Levels of expression for the majority of identified genes differed among species and gene expression levels (FPKM values) were ranked in order from the highest to lowest as *M*. *novaehollandiae* > *M*. *australiense* > *M*. *tolmerum* (e.g., see fig. 4 and supplementary fig. S3, Supplementary Material online). In general, gene expression is highly plastic and can be influenced strongly by a variety of environmental factors (Nadal et al. 2011); plasticity in gene expression to a common environmental change can differ among different lineages and populations (Kozak et al. 2014; Kavembe et al. 2015). The three *Macrobrachium* species compared here come from distinct phylogenetic lineages and are found in different natural salinity conditions in the wild. When individuals of each taxa were exposed to the same osmotic conditions, the same set of functional genes and transcripts were expressed, but at very different levels (large differences in FPKM values between 0‰ and 15‰) in each species. These results clearly indicate the important functional roles of these candidate genes in dealing with changes to osmotic conditions. Therefore, it is conceivable that these genes would have played key roles during the transition from marine/brackish water to freshwater by ancestral forms.

As a brackish-water inhabitant over its entire life cycle (where maintaining ionic balance is less stressful/energetically demanding compared with freshwater) (Faleiros et al. 2010), *M*. *novaehollandiae* has the advantage of experiencing almost constant iso-osmotic conditions with the surrounding environment. Thus, osmoregulation for *M*. *novaehollandiae* may be more difficult and likely requires higher energy expenditure in freshwater. This probably explains why the highest number of differentially expressed transcripts (925), and higher expression levels (FPKM values) of genes/transcripts were observed for *M*. *novaehollandiae* when all test individuals were exposed to 0‰. *Macrobrachiumtolmerum* in contrast showed comparatively lower levels of gene expression and the lowest number of differentially expressed transcripts (861). This probably reflects natural regular dispersal by *M*. *tolmerum* between marine and freshwater environments (larval development occurs in sea water) where tolerance of a wide range of natural salinity fluctuations is required. *Macrobrachiumaustraliense* is well adapted to freshwater environments and any increase in salinity likely imposes a significant level of osmotic stress for this species but not to the same degree as for *M*. *novaehollandiae* in freshwater. Here, we observed only moderate levels of gene expression and also a lower number of differentially expressed transcripts (876) for *M*. *australiense*.

### Differential Expression Pattern of Novel Transcripts

We identified 16 unannotated putatively novel transcripts/genes that were differentially expressed between salinities in all three species (supplementary fig. S3 and supplementary table S4, Supplementary Material online). This suggests that they potentially have important functional roles in adaptation to different osmotic niches. Gene duplication, loss and/or gain are common events during evolutionary change and lineage splitting in nature (Lynch and Conery 2000; Colbourne et al. 2011; McLysaght and Hurst 2016). Taxonomically restricted novel (orphan) genes are in general, smaller in length, quicker to become functional, are generally expressed at higher levels and then reduce expression levels or even can become pseudo-genes when adaptation is complete (Schlotterer 2015). Expression levels of orphan genes also determine their stability, whereas highly expressed orphan genes generally persist longer and may even become conserved (McLysaght and Hurst 2016). Conserved orphan genes tend to increase in expression level with increased age (Palmieri et al. 2014). The *Macrobrachium* lineage may have gained these 16 novel coding sequences at different stages during their evolutionary origin, and potentially these may have occurred during the late Oligocene or early Miocene periods (at least 6.5 Ma) when this genus first evolved (Murphy and Austin 2005; Wowor et al. 2009; Rahi et al. 2017). The 16 orphan transcripts identified here are all highly expressed in the three target species, suggesting that they could be quite old and play important functional roles in *Macrobrachium* taxa. Because the genus *Macrobrachium* is very distantly related to *Daphnia*, it is difficult to determine if the 16 novel orphan transcripts identified here in *Macrobrachium* taxa are specific to this lineage or may be present more widely in some other/all decapod taxa. As many crustacean transcriptome are now publicly available, more species can be sequenced and integrated with the existing lineages to determine the age of lineage-specific orphan, or taxonomically restricted, genes in the future.

### Signatures of Selection Acting on Candidate Genes

Out of all the genes studied here (205 differentially expressed and 43 preidentified), only 26 genes showed evidence for signatures of historical positive selection (table 5 and supplementary table S5, Supplementary Material online). Of note however, was that the majority of the genes with osmoregulatory function (including genes that control epithelial permeability and cell volume regulation) did not show evidence of positive selection in pairwise species comparisons. The implication is that genes involved with ionic balance in *Macrobrachium* taxa are most probably highly conserved. The major challenge for an organism in any aquatic environment is to maintain ionic balance which is achieved via osmoregulatory processes (Henry et al. 2012; McNamara et al. 2015). Thus, osmoregulatory genes are likely to be exposed to the same type of strong selection pressures across taxa (pressure to maintain efficient ion transport and exchange to facilitate internal iso-osmotic conditions) in any aquatic environment (fresh, brackish or marine waters). This may explain why we observed evidence for action of purifying selection (*d*_N_/*d*_S_ < 1) on candidate osmoregulatory genes in all three *Macrobrachium* species. Moreover, osmotic gradients can change in any aquatic environment and can vary depending on season, so organisms periodically face ionic fluctuations, at least to some extent (Zhu et al. 2016). Candidate genes need to respond immediately for regulatory cell volume increase or decrease to cope with changes to the surrounding environment (Henry et al. 2012; Havird et al. 2013) and thus, are likely to be exposed to the action of purifying selection. High sequence conservation of osmoregulatory genes may also indicate that these genes are, in relative terms, quite primitive and likely evolved first in a common marine ancestor to maintain ionic balance, whereas in modern taxa they still perform essentially the same functional roles in both brackish and freshwater adapted species.

Of note, we found strong signatures of positive selection on only four osmotic stress response genes (*Calreticulin*, *Interleukin*, *P38 MAP kinase*, and *Selanophosphate*) in comparisons between ALD and ELD species. One possible explanation for this result is that ALD species are unlikely to experience salinity stress over their life cycle, whereas ELD species frequently experience changes to environmental salinity over their life cycle. Thus, different aquatic habitats can impose completely different types of ionic stress on species with different life history patterns (Mitterboeck et al. 2016). Low ionic freshwater environments impose stress at a different order of magnitude compared with highly ion rich saline water, and individual species inhabiting different environmental conditions are well adapted to each corresponding habitat. Thus, functional roles of the four osmotic stress response genes identified here may be very specific to each corresponding habitat (Barman et al. 2012; Havird et al. 2013). We consider that this could potentially explain the reason for higher sequence divergence in genes involved with osmotic stress tolerance in different ALD and ELD *Macrobrachium* taxa examined here.

We also observed strong positive selection on genes that maintain water balance and cellular junctions (*Aquaporin*, *Claudin*, and *Integrin*). Tighter cell junctions are required for osmoregulation in freshwater compared with raised salinity conditions (Tipsmark et al. 2002; Brennan et al. 2015; Velotta et al. 2017). The intensity of selection pressure acts differently under different environmental conditions that in turn can result in sequence divergence in the coding regions in a number of candidate genes controlling a specific phenotype while maintaining the sequences conserved in other genes (Smith and Eyre-walker 2002; Ellegren 2014; Berdan et al. 2015; Bailey and Bataillon 2016). Thus, differences in environmental conditions impose different types of selective pressure on underlying genes of ALD versus ELD species. Over time, this selection pressure should lead to accumulation of sequence variation in certain genes that facilitates efficient adaptation to different osmotic conditions. In combination, differential effects of selection on seven genes (*Aquaporin*, *Claudin*, *Integrin*, *Calreticulin*, *Interleukin*, *P38 MAP kinase*, and *Selanophosphate*) in ALD versus ELD species provide strong evidence for the impact of freshwater adaptation.

In a diverse array of crustacean species, regulation of internal body fluid content via hemolymph production is considered to be one of the principal mechanisms for maintaining an iso-osmotic body fluid concentration relative to the surrounding aquatic medium (McNamara et al. 2015; Mitterboeck et al. 2016). Three master genes (*Crustacean cardiovascular peptide*, *Diuretic hormone*, and *Hyperglycaemic hormone*) are known to be key drivers of this process in crustaceans (Rahi et al. 2018). Both *Hyperglycaemic hormone* and *Diuretic hormone* showed signatures of positive selection in the three *Macrobrachium* taxa investigated here, indicating adaptive sequence divergence of these genes over a prolonged time period as a response to exposure to different osmotic conditions.

Of all the genes studied here, the strongest evidence for positive selection was observed in genes that are associated with controlling egg size and larval developmental stages. Eight genes out of 11 in these categories showed strong signals of positive selection. Genes that control egg number and egg size, and also number of larval developmental stages, did not show salinity induced differential expression patterns in any species. This result, however, does not mean that these genes are not important for freshwater adaptation because there are clear differences among these traits (egg number, size, and larval stage number) between ALD and ELD *Macrobrachium* taxa (Vogt 2013). Modification of expression patterns of these genes may not be related to direct exposure to different osmotic environments. Rather, changes likely resulted from historical functional mutations in the candidate genes controlling these phenotypes. There is evidence that evolution of many traits did not occur by changes to gene expression or gene regulation (Romero et al. 2012). Thus, these genes may be under selective pressure from other factors inherent to a freshwater environment and not directly to differing osmotic niches. As an example, changes to egg number, egg size, and number of larval stages may not be prerequisites for initial invasion of freshwater but rather may arise via parallel evolution after successful colonization of freshwater. These traits potentially are linked to a need to cope with unidirectional water flow in freshwater; direct development of larvae therefore is favored because they need to negate the effects of a net downstream displacement.

Potentially, adaptive mutations in genes that affect egg size and larval development may have evolved after ALD species had successfully colonized freshwater habitats and may reflect an evolutionary trade-off between freshwater adaptation and producing a few large eggs with reduced number of developmental stages. Adaptation to new environments via novel mutations requires significant evolutionary time, but once the mutations occur, they can bring major and rapid phenotypic change (Baldo et al. 2011; Wray 2013; Berens et al. 2015). We believe that in the current study, this may explain the reason for higher sequence divergence between ALD and ELD species and absence of differential expression patterns in genes involved with egg size and larval developmental stage in the *Macrobrachium* taxa. None of the 16 novel transcripts showed signs of positive selection and sequences were highly conserved (*d*_N_/*d*_S_ < 0.5) among the species studied. Only nine genes (*α-amylase*, *Growth-arrest-protein*, *RAP4*, *Heparan sulfate 6*, *Pantothanate flavoprotein*, *Glutatheone synthetase*, *Syndecan 2*, *TKT protein*, and *UbiquitinC*) from the remaining other functional categories (genes for growth, feeding, energetics, stress response, and metabolic processes) showed the impact of positive selection. It clearly indicates that these nine genes are also important candidate genes for freshwater adaptation in different *Macrobrachium* lineages.

### Overlap between Gene Expression and Sequence Divergence

Overall, we observed discordance in overlap between sequence divergence (effects of positive selection) and differential expression patterns in most of the genes assessed here. This pattern may result from three alternate processes. First, adaptation to freshwater may require fine-tuning (sequence variation/divergence) of certain existing candidate genes and changes to expression patterns (plasticity) of other underlying genes (Wray 2013; Kozak et al. 2014). Second, physiological divergence may occur between species during freshwater invasion (Kozak et al. 2014), and the adaptation process may be compensated for by plasticity in gene expression patterns and existing standing genetic variation (Stapley et al. 2010). Third, functional mutations over a prolonged evolutionary timescale may provide sufficient support for efficient adaptation which in turn, may bring some changes in phenotypic traits (e.g., egg size and larval stage numbers). Overlap between sequence divergence and higher expression patterns (FPKM values) were detected in only nine candidate genes (out of all genes examined here), indicating very important functional roles of these genes under different osmotic conditions.

One question arising from the results of the study was: “did larger eggs and other ALD specific traits facilitate freshwater adaptation by specific *Macrobrachium* taxa?” or “do these traits potentially represent the consequences of freshwater adaptation?” At this stage, we argue that these traits are more likely to be the consequences of freshwater adaptation. We offer three potential explanations to support this hypothesis. First, none of these traits have been described previously in any brackish-water or marine *Macrobrachium* species, and not even in closely related marine taxa (Vogt 2013). Second, eggs of marine species are more prone to water loss and it is difficult to retain water in larger eggs; thus small eggs are more likely to constitute the ancestral (marine) trait. Moreover, differences exist in the levels of relative productivity between fresh and sea water with most freshwater having lower levels of primary production, so, larvae of ALD species require sufficient nutrition to support their development in freshwater which they can access from large yolky eggs. Third, ALD species may have gained these traits as an evolutionary trade-off so that hatchlings can actively swim against fast flowing water in freshwater streams; otherwise, larvae are likely to face downstream displacement and may die due to increasing salinity, temperature, or other nonfavored conditions as they approach the sea.

## Conclusions

Freshwater adaptation is a complex process that involves multiple interactions among many interacting genes. In essence, it may occur via an “independent-weaker/interaction-stronger effect” process where individual genes have little impact in isolation, but in combination with many other genes (epistasis) can allow efficient adaptation. The same genes at the same time may have multiple functional roles. In the current study, 205 candidate genes were identified besides 43 preidentified genes that are likely to be involved with freshwater adaptation in different *Macrobrachium* species. The same genes and same molecular mechanisms are likely to be involved with freshwater adaptation in *Macrobrachium* species (broadly in aquatic crustaceans) and are also likely to be conserved via the same molecular pathways. Traits that are the consequences of freshwater adaptation likely include evolution of large eggs and direct development in contrast to numerous small eggs with many larval developmental stages. Relatively high number of differentially expressed genes in parallel with a relatively lower number of genes under positive selection indicates that the adaptive response to freshwater environments was more likely to be driven initially by changes in gene expression patterns in a large number of genes and genetic modifications to only a small number of genes. Differentially expressed genes, novel sequences and positively selected genes identified here provide potential candidates for future studies that can more fully document the molecular basis of freshwater adaptation in aquatic crustaceans. In addition, epigenetic mechanisms may also have played an important supportive role in freshwater adaptation in crustaceans and this possibility provides further experimental opportunities to investigate.

## Data Archiving

This project has been deposited at NCBI (SRA and TSA) under the accession numbers: SAMN09435338, SAMN09435339 and GHDT00000000 (Macrobrachium australiense), SAMN09435342, SAMN09435343 and GHDQ00000000 (Macrobrachium tolmerum), SAMN09435340, SAMN09435341 and GHDW00000000 (Macrobrachium novaehollandiae), and SAMN09435344, SAMN09435345 and GHDT00000000 (Macrobrachium koombooloomba). The datasets are also available at QUT Library Research Data Repository Under the DOI: org/10.4225/09/597ec66cdd454.

## Supplementary Material

Supplementary data are available at *Genome Biology and Evolution* online.

## Supplementary Material

Supplementary Data

## Notes

**Data deposition:** This project has been deposited at NCBI (SRA and TSA) under the accession numbers: SAMN09435338, SAMN09435339 and GHDT00000000 (Macrobrachium australiense), SAMN09435342, SAMN09435343 and GHDQ00000000 (Macrobrachium tolmerum), SAMN09435340, SAMN09435341 and GHDW00000000 (Macrobrachium novaehollandiae), and SAMN09435344, SAMN09435345 and GHDT00000000 (Macrobrachium koombooloomba). The datasets are also available at QUT Library Research Data Repository Under the DOI: org/10.4225/09/597ec66cdd454.
