# Supplementary material for: The Molecular Basis of Freshwater Adaptation in Prawns: Insights from Comparative Transcriptomics of Three Macrobrachium Species
Source: Genome Biol Evol. 2019 Mar 6;11(4):1002–18. doi: 10.1093/gbe/evz045 (PMC6450038; doi:10.1093/gbe/evz045)
Supplement: Supplementary Data [file evz045_supp.docx]

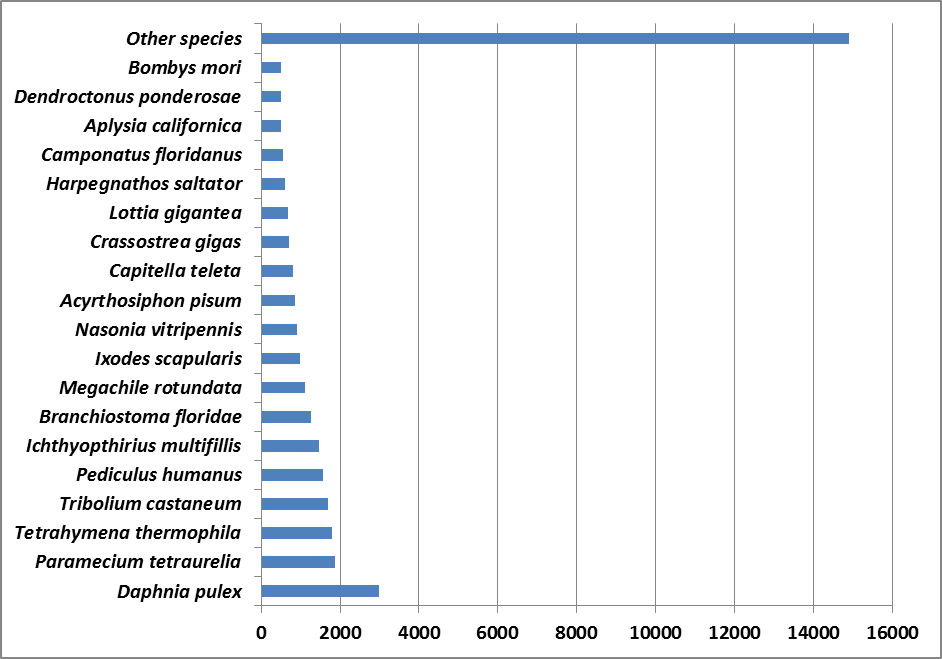

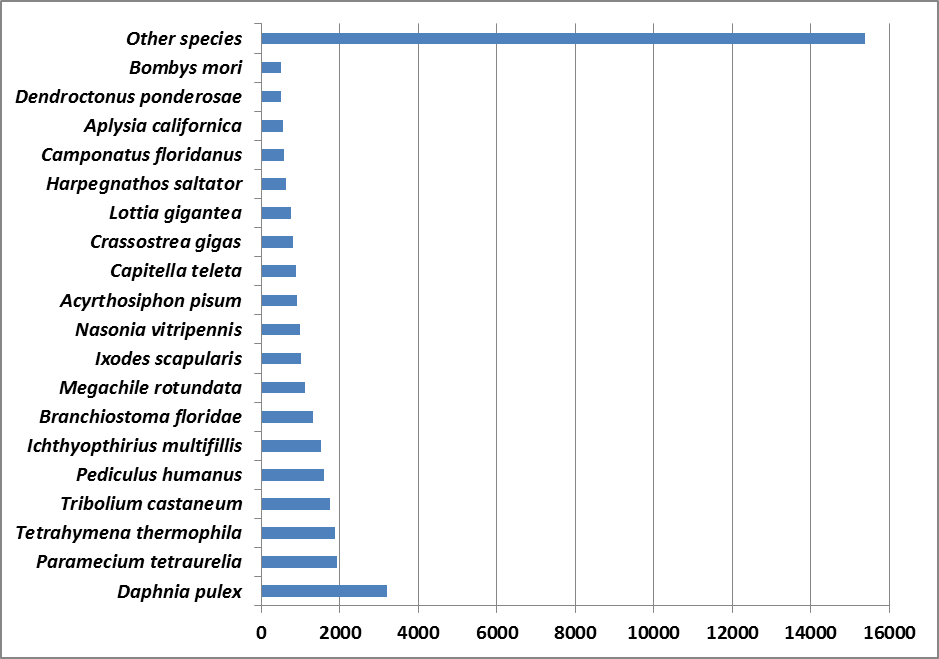

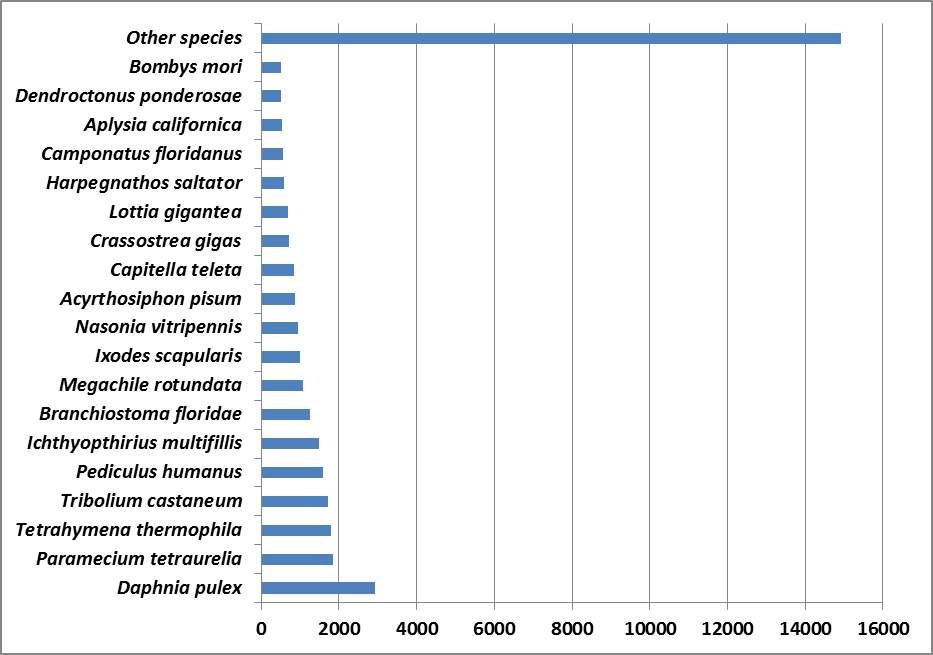


**a**

**b**

**c**

**Fig. S1.** Top hit species distribution chart: a) *M*. *australiense*, b) *M*. *tolmerum* and c) *M*. *novaehollandiae*. X-axis represents the number of blast matches while Y-axis shows the name of different species, other species includes blast matches with all other existing species in public database.


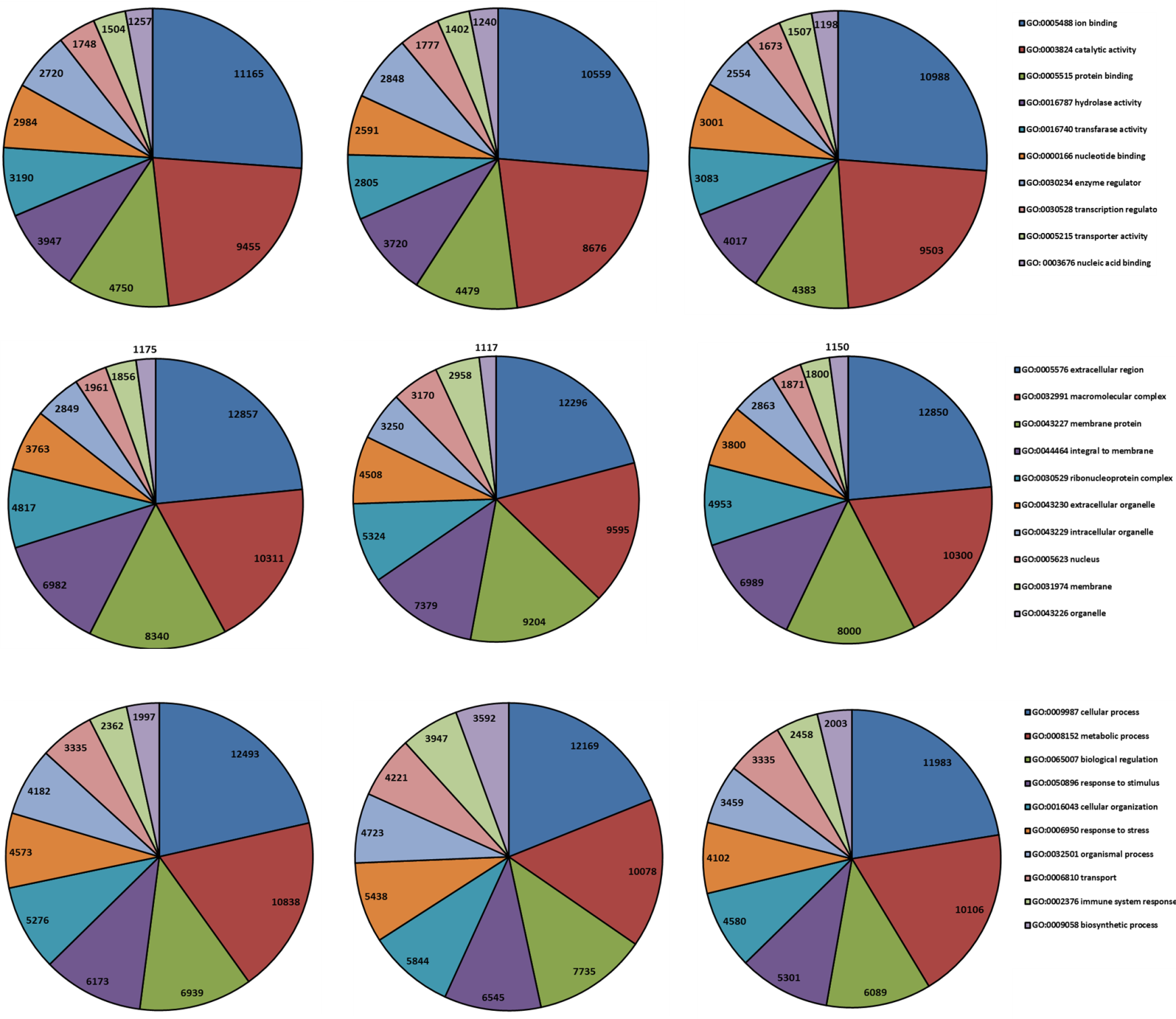


a 1

a 2

a 3

b 1

b 2

b 3

c 1

c 2

c 3

**Fig. S2.** Top most abundant GO categories for each species: a) Cellular component, b) Molecular functions, c) Biological processes. 1 = *M*. *australiense*, 2 = *M*. *tolmerum*, 3 = *M*. *novaehollandiae*.


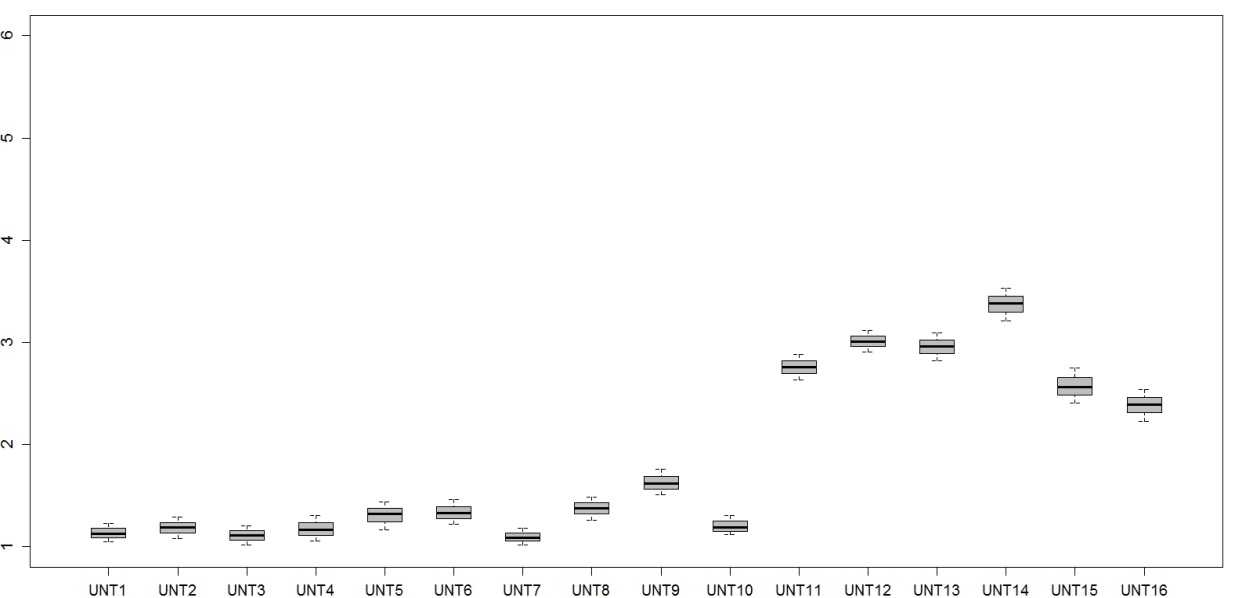

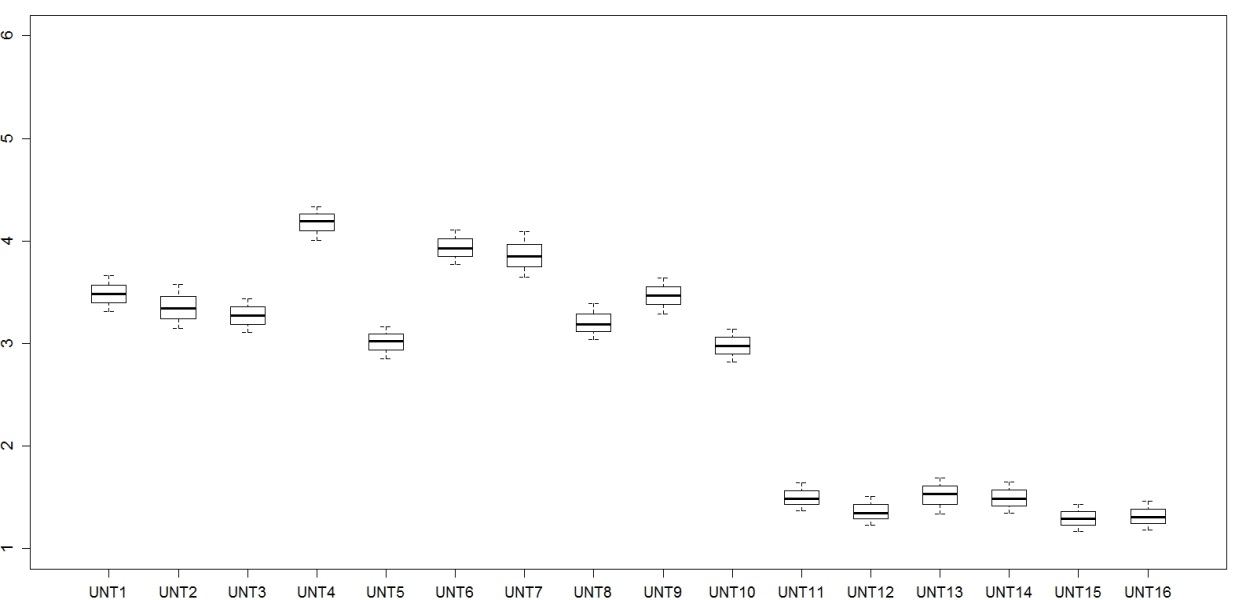


a


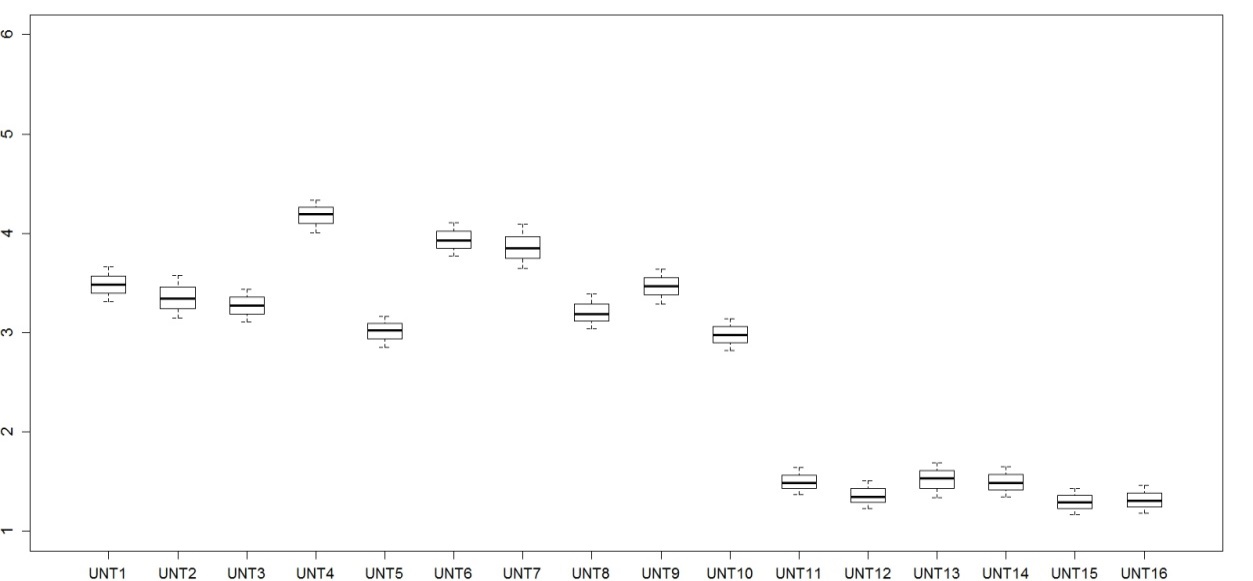

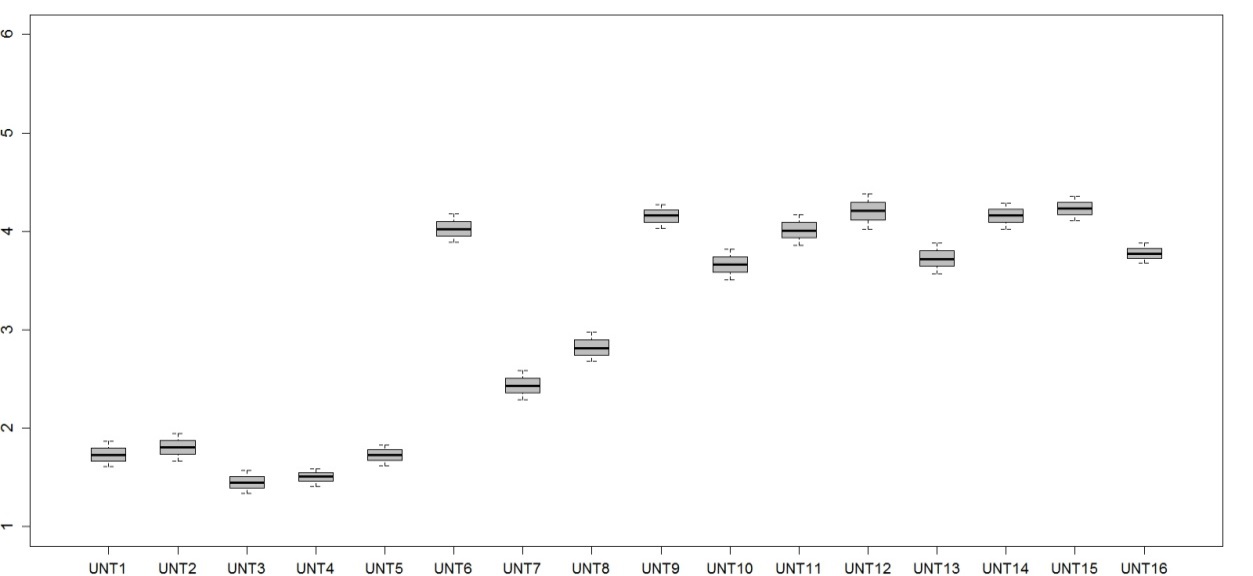

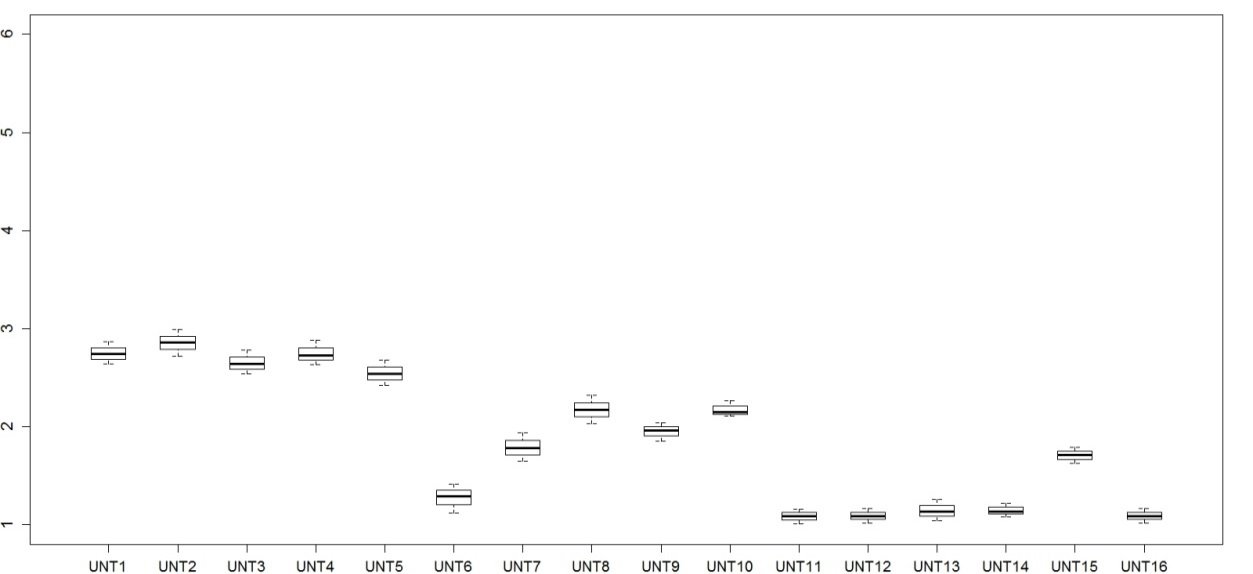

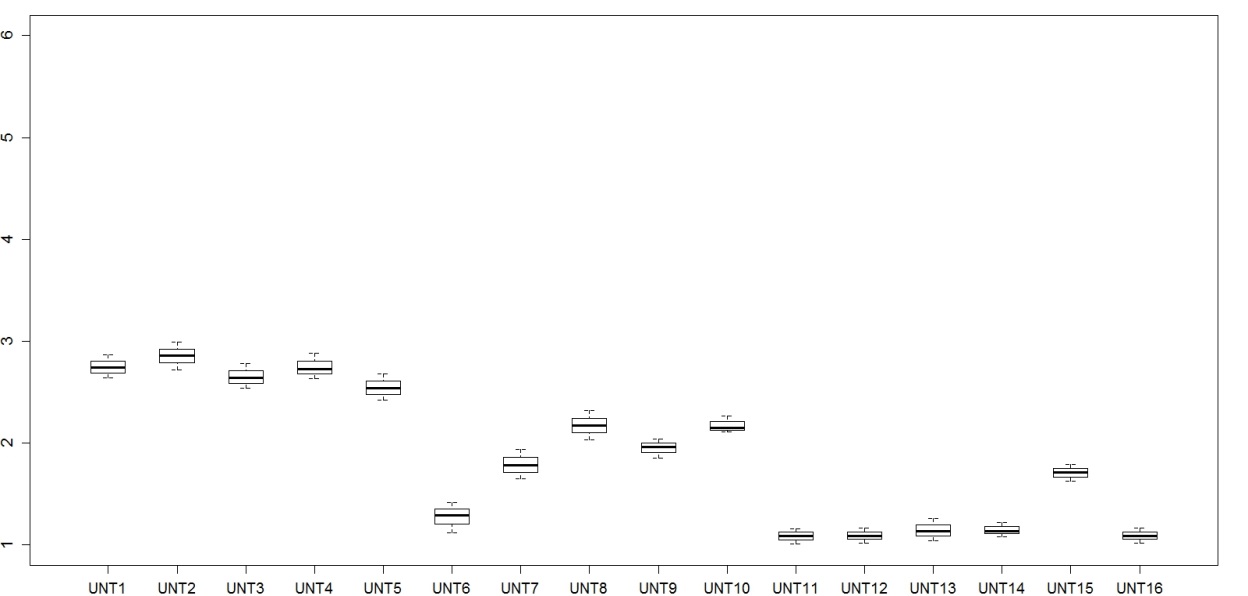

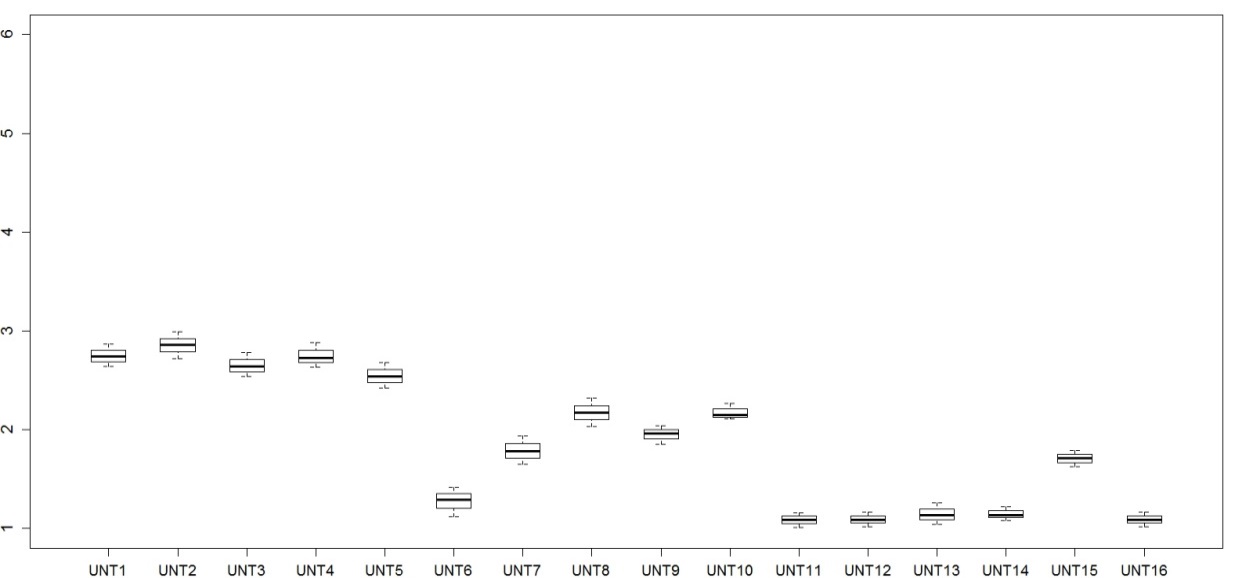


b


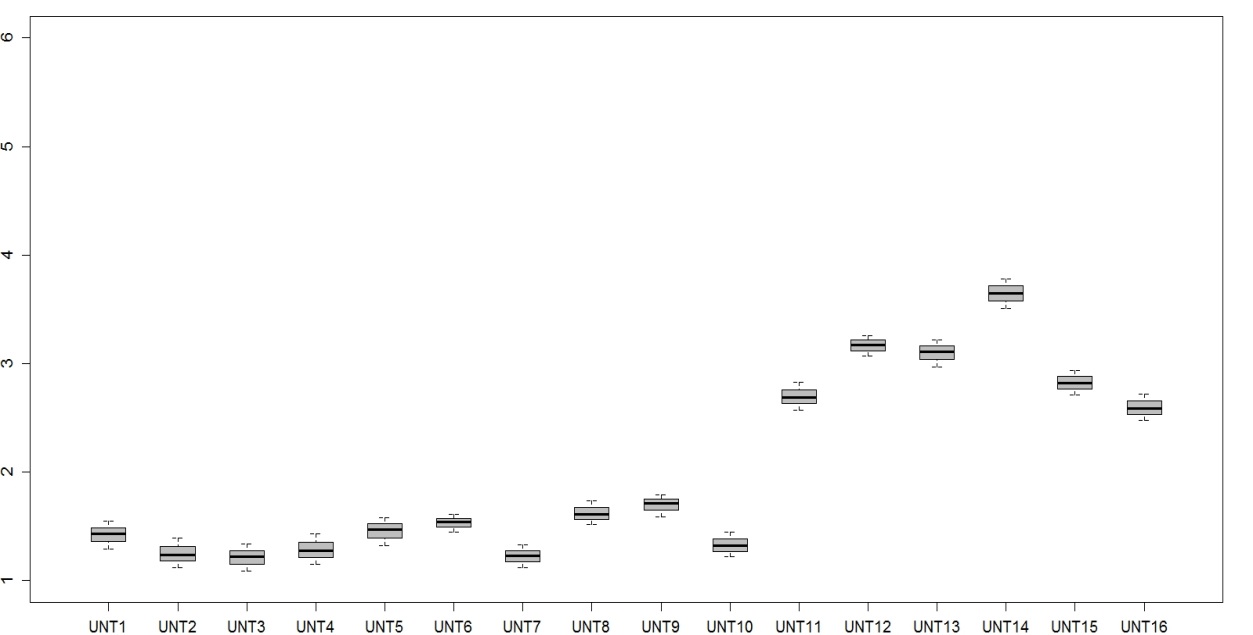

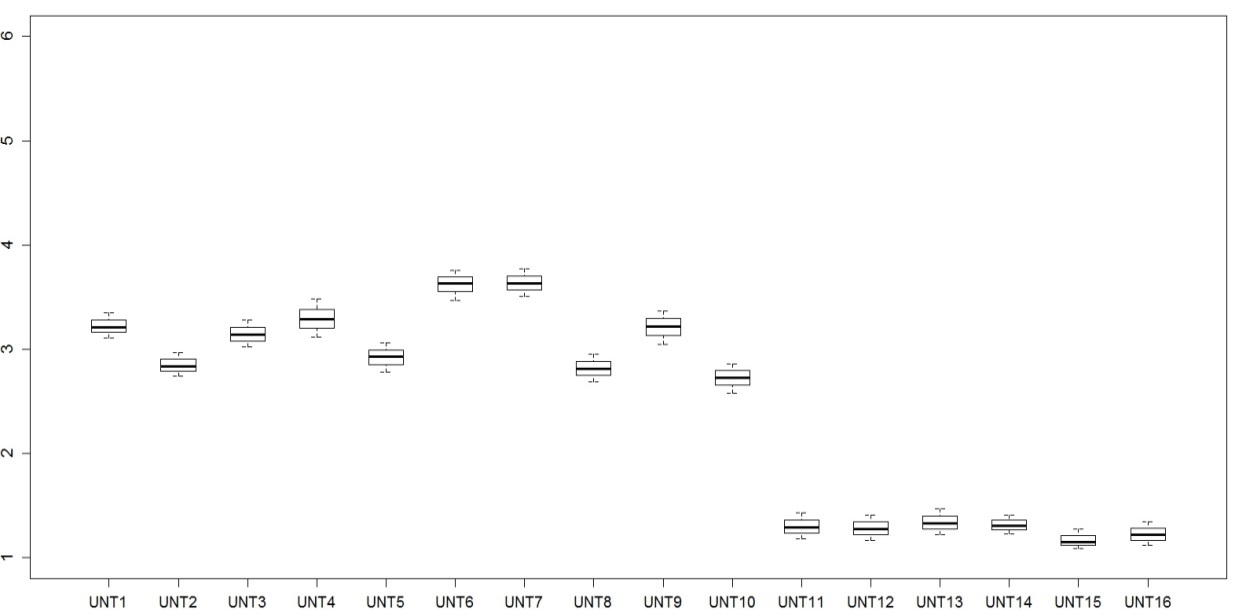

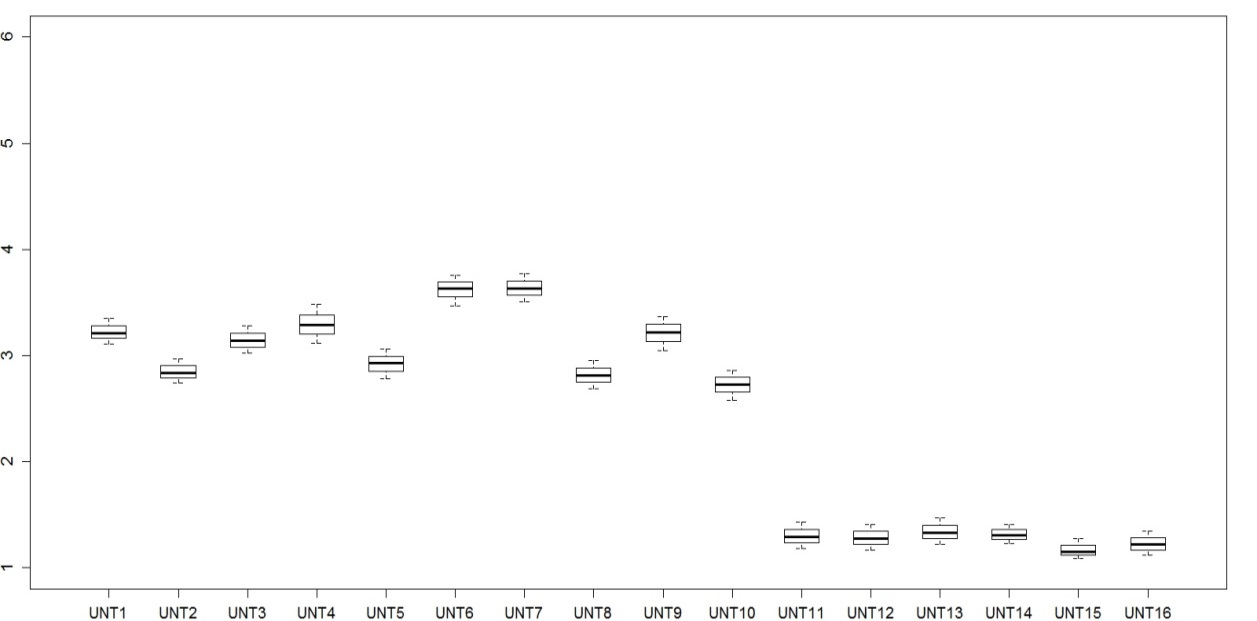


c

Expression Pattern in Log(FPKM)

Expression Pattern in Log(FPKM)

Expression Pattern in Log(FPKM)

**Fig. S3.** Box and Whisker plots showing variation in differential expression patterns of 16 novel transcripts at 0‰ (grey boxes) and 15‰ (white boxes): a) *M*. *australiense*, b) *M*. *novaehollandiae* and c) *M*. *tolmerum*. UNT = Unknown Transcript


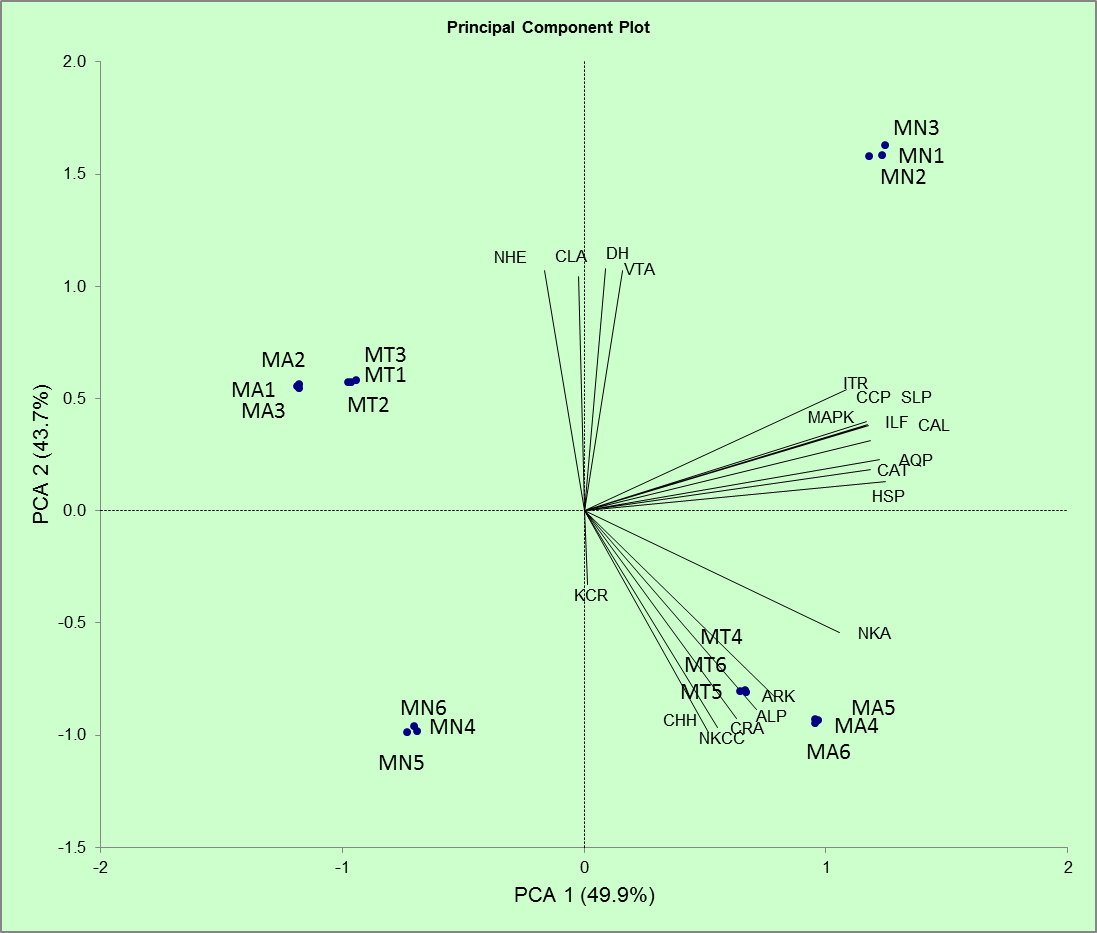


**Fig. S4.** PCA plot for the top most differentially expressed 20 pre-identified genes of the three *Macrobrachium* species: MA = *M*. *australiense*, MN = *M*. *novaehollandiae* and MT = *M*. *tolmerum*. 1, 2 & 3 indicate individuals from 0‰ while 4, 5 & 6 indicate individuals from 15‰.


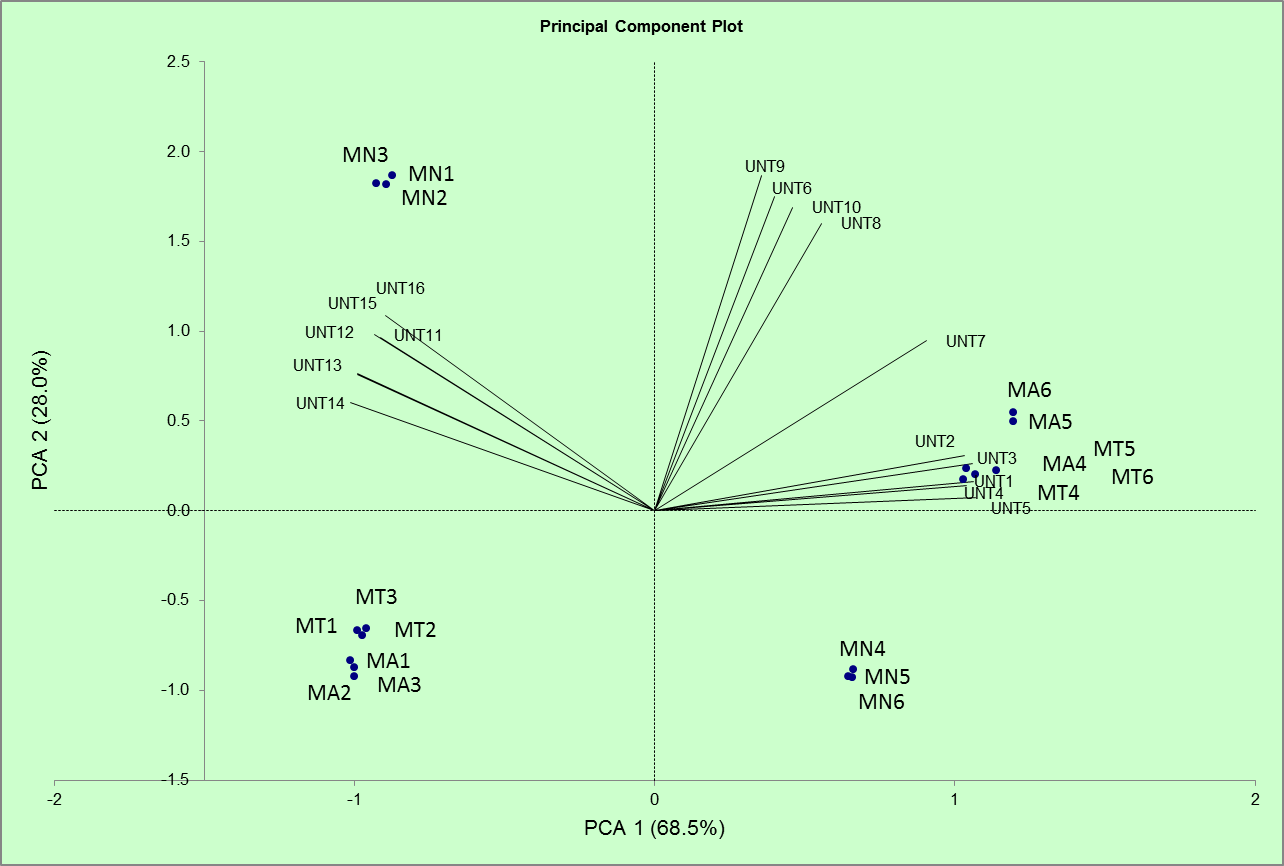


**Fig. S5.** PCA for the 16 novel transcripts of the three *Macrobrachium* species: MA = *M*. *australiense*, MN = *M*. *novaehollandiae* and MT = *M*. *tolmerum*. 1, 2 & 3 indicate individuals from 0‰ while 4, 5 & 6 indicate individuals from 15‰.


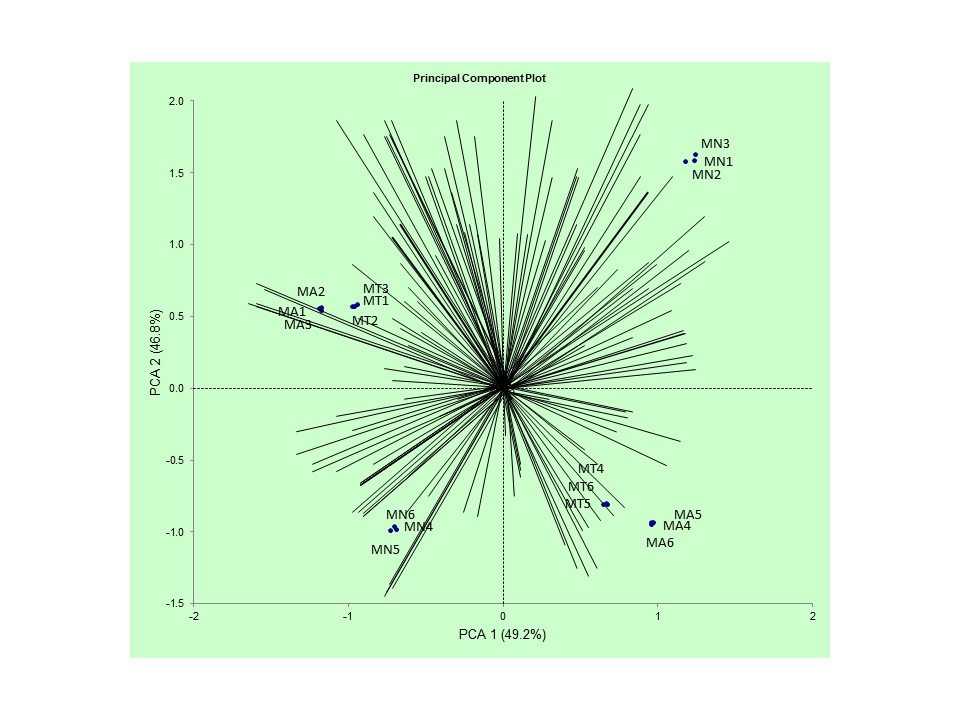


**Fig. S6.** PCA plots of 783 commonly differentially expressed transcripts for the three *Macrobrachium* species: MA= *M*. *australiense*, MT= *M*. *tolmerum* and MN= *M*. *novaehollandiae*. 1, 2 & 3 indicate individuals from 0‰ while 4, 5 & 6 indicate individuals from 15‰.


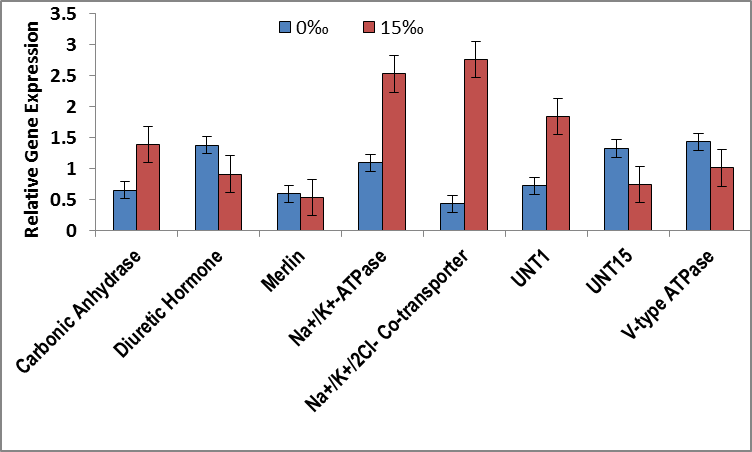

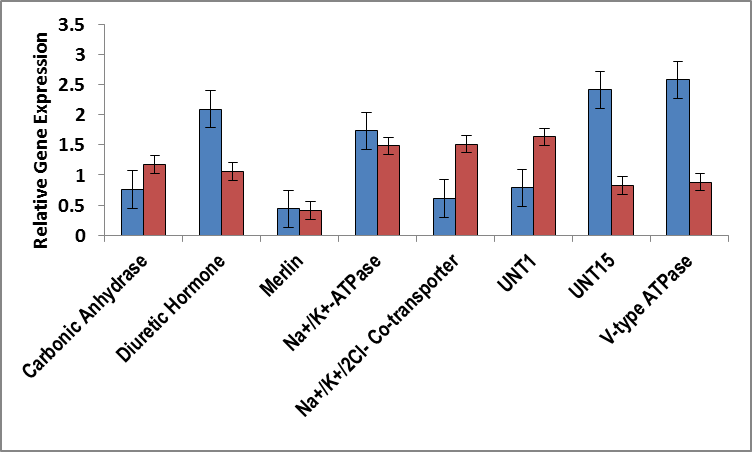

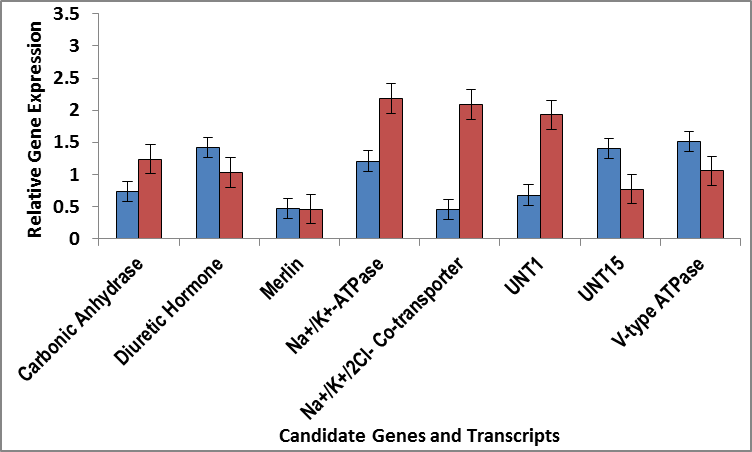


**a**

**b**

**c**

**Fig. S7.** RT-qPCR based relative expression pattern of candidate genes and transcripts. X-axis represents the candidate genes and transcripts while Y-axis is showing the relative expression pattern (error bars represent +/-1 SE). a) *M*. *australiense*, b) *M*. *novaehollandiae*, c) *M*. *tolmerum*

**Table S1** Candidate genes involved with freshwater adaptation in a range of crustacean species.

| **Gene Name** | **Functional Role** | **References** |
| --- | --- | --- |
| Alkaline Phosphatase | Phosphatase activity, ion binding and precipitation | Pongsomboon et al. 2009; Moshtaghi et al. 2016 |
| Aquaporin | Ion transport, cell volume regulation, water channel regulation | Barman et al. 2012; Rahi et al. 2017 |
| Arginine Kinase | ATP binding, ion transport and salinity regulation | Henry et al. 2012; Ali et al. 2015 |
| ABC family | ATP binding, ATPase activity, ion transport, stress response | Barman et al. 2012; Rahi et al. 2017 |
| Ca^+2^/Cl^-^ channel regulator | Protein binding, ion channel regulation, Ca^+2^ and Cl^-^ transport | Havird et al. 2013; Rahi et al. 2017 |
| Ca^+2^/Cl^-^ channel precursor | Ion transport and protein binding | Rahi et al. 2017 |
| Ca^+2^-ATPase | Ca^+2^ ion: binding, transport and regulation, calcification | Ali et al. 2015; Moshtaghi et al. 2016 |
| Cbl | Cell signaling, protein ubiquitination, oogenesis | Colbourne et al. 2007; Rahi et al. 2017 |
| Calreticulin | Osmotic signal transduction, response to osmotic stress | Barman et al. 2012; Ali et al. 2015 |
| Carbonic Anhydrase | pH balance, ion binding and exchange, transferase activity | Ali et al. 2015; Moshtaghi et al. 2016 |
| Claudin | Maintain cellular junction and epithelial permiability | Furriel et al. 2010; Kozak et al. 2013 |
| Crustacean cardiovascular peptide | Stress tolerance, signaling, haemolymph production and body fluid maintenance | Berkefeld et al. 2010; Rahi et al. 2017 |
| Crustacean hyperglycemic hormone | Stress signaling pathway, haemolymph production | Moshtaghi et al. 2016; Rahi et al. 2017 |
| Cullin | Cellular processes, developmental roles, egg production | Colbourne et al. 2011 |
| Diuretic Hormone | Water balance, haemolymph balance | Chen et al. 2015 |
| Heat shock proteins | Stress response, chaperone function, protein folding | Tongsaikling et al. 2013; Chen et al. 2015 |
| H^+^/Cl^-^ exchanger | Regulate Cl^-^ channel & cell volume, ion transport | Ali et al. 2015; Moshtaghi et al. 2016 |
| Integrin | Regulate cellular volume & junction, mediate signal pathways | Barman et al. 2012; Rahi et al. 2017 |
| ILF2 | ATP binding, salinity stress signalling | Henry et al. 2012; Chen et al. 2015 |
| K^+^Cl^-^ symporter | Integral membrane component, ion channel regulatory activity | Pongsomboon et al. 2009 |
| Leukocyte ARL | Oogenesis, cellular morphogenesis | Colbourne et al. 2011; Rahi et al. 2017 |
| P38 MAP Kinase | Osmotic signal transfer, phosphorylation, ATP binding | Barman et al. 2012; Kozak et al. 2013 |
| Mastermind | Embryogenesis, larval development, DNA binding | Colbourne et al. 2011; Rahi et al. 2017 |
| Merlin | Developmental role, maintain developmental duration | Mensch et al. 2008; Harney et al. 2015 |
| Midline | Multiple developmental roles, embryogenesis | Mensch et al. 2008; Harney et al. 2015 |
| Mitochondrial carrier protein | Ion transport, osmotic signal transduction, transmembrane transport, membrane component | Yang et al. 2011 |
| Mothers against DPP | Gastrulation, morphogenesis, egg development | Colbourne et al. 2007; Rahi et al. 2017 |
| Mg^+2^ Transporter | Ma^+2^ transportation, integral membrane component | Leite and Zanotto 2013 |
| Na^+^/K^+^ ATPase | Ion exchange & binding, ion balance, transmembrane transport | Faleiros et al. 2010; Ali et al. 2015; Chen et al. 2015; Moshtaghi et al. 2016 |
| Na^+^/HCO_3_^-^ transporter | ATP binding, transport and exchange of anions | Havird et al. 2013 |
| Na^+^/Ca^+2^ exchanger | Ca^+2^ transport, Na^+^/Ca^+2^ antiporter activity, cell communication | Ali et al. 2015; Rahi et al. 2017 |
| Na^+^/Cl^-^ Cotransporter | Drive ion exchange, generate electrochemical gradient | Velotta et al. 2015 |
| Na^+^/K^+^/2Cl^-^cotransporter | Na^+^:K^+^:2Cl^-^ symporter activity, ion transport and exchange, integral membrane component | Faleiros et al. 2010; Barman et al. 2012; Ali et al. 2015; Moshtaghi et al. 2016 |
| Na^+^/H^+^ exchanger | Na^+^& H^+^ transport, Na^+^:H^+^ antiporter activity, absorb Na^+^ | Havird et al. 2013; Moshtaghi et al. 2016 |
| Na^+^/K^+^/Ca^+2^ exchanger | Ca^+2^/Na^+^:K^+^ antiporter activity, ion transport | Rahi et al. 2017 |
| Na^+^ transporter | Transmembrane transporter, transporter activity | Ali et al. 2015 |
| Potassium Channel | K^+^ transmembrane transport, K^+^ channel activity | Pongsomboon et al. 2009 |
| Selenophosphate | ATP binding, osmotic and oxidative stress response | Barman et al. 2012; Moshtaghi et al. 2016 |
| Serpin | Larval development, chaperone & storage functions | Colbourne et al. 2011; Rahi et al. 2017 |
| V (H^+^) ATPase | Pumps H^+^ in dilute medium for ionic balance, intake ions | Ali et al. 2015; Moshtaghi et al. 2016 |
| Plekstrin homology domain protein | Intracellular signaling, membrane protein and cellular component, egg production | Rahi et al. 2017 |
| Vitelline membrane outer layer protein | Integral membrane protein, egg size | Berkefeld et al. 2010 |
| Vitellogenin | Oogenesis, nutrient reservation and lipid transport | Tongsaikling et al. 2013 |

**Table S2** Specific primers for RT-qPCR assay to validate differential gene expression pattern.

| **Gene name** | **Primer type** | **Sequence** | **Tm (⁰C)** | **Product size (bp)** |
| --- | --- | --- | --- | --- |
| Diuretic horomne | Forward | ACAACTCGGCTCTGGTGTTC | 60 | 224 |
|  | Reverse | CTCGGCCTAGACCCAAGTC | 59 |  |
| Merlin | Forward | CAAAGCATTTGTTGCTGAACAC | 61 | 211 |
|  | Reverse | TACCAGGGTGGCTTTCTTTG | 60 |  |
| Na^+^/K^+^-ATPase | Forward | CACCCCACCCAAACAAACT | 60 | 219 |
|  | Reverse | TCGTGAACTCTTGCTTTCTTGA | 60 |  |
| Na^+^/K^+^/2Cl^-^ Cotransporter | Forward | GGGTCACCAGGGTCCAGAT | 59 | 180 |
|  | Reverse | TAGCACCAGCAACAATTCCA | 54 |  |
| Carbonic Anhydrase | Forward | CATCATGAGGACGGCCATAG | 61 | 203 |
|  | Reverse | TCCCAGTCTCTGAGAAGCAGA | 60 |  |
| UNT1 | Forward | CAGCAGCAAAGGCAGCAG | 62 | 164 |
|  | Reverse | TGCAGCAGTGGCTCTCTCTA | 59 |  |
| UNT15 | Forward | TGCCAACAACTATGCCAATG | 61 | 201 |
|  | Reverse | CCGAATCCACCGTTCAAG | 58 |  |
| V-type H^+^ ATPase | Forward | GTCGATTTTCCGAAGCGAAT | 62 | 205 |
|  | Reverse | GCCTTCCAAGTAGCGAAGC | 60 |  |
| 18S | Forward | GCGGTAATTCCAGCTCCA | 55 | 200 |
|  | Reverse | AGCCTGCTTTGAGCACTCTC | 58 |  |

**Table S3** Some important candidate genes (pre-identified 43 and 16 novel transcripts) with GO-terms involved with freshwater adaptation in the three *Macrobrachium* species.

| **Contig ID** | | | **Gene Name** | **Gene Length (amino acids)** | **GO-terms** |
| --- | --- | --- | --- | --- | --- |
| *M*. *australiense* | *M*. *tolmerum* | *M*. *novaehollandiae* |  |  |  |
| c57163_g1_i2 | c61758_g1_i1 | TR51270\|c0_g1_i6 | Alkaline Phosphatase | 560 | Ion binding |
| c58766_g7_i1 | c72824_g1_i1 | TR49913\|c0_g1_i3 | Aquaporin | 303 | Cell volume |
| c58721_g2_i2 | c66845_g1_i2 | TR43784\|c0_g8_i2 | Arginine Kinase | 630 | Ion transport |
| c58392_g3_i2 | c71615_g1_i4 | TR52129\|c0_g1_i2 | ABC Family | 2010 | Osmotic stress |
| c59448_g3_i1 | c69623_g2_i1 | TR50500\|c0_g1_i1 | Ca^+2^/Cl^-^ channel regulator | 625 | Ion channel |
| c59448_g3_i2 | c64477_g1_i1 | TR52006\|c0_g1_i2 | Ca^+2^/Cl^-^ channel precursor | 959 | Ion transport |
| c58132_g1_i4 | c67478_g1_i1 | TR35544\|c2_g1_i4 | Ca^+2^-ATPase | 1020 | Ion regulation |
| c50036_g2_i2 | c55819_g1_i2 | TR79893\|c0_g1_i1 | Cbl | 185 | Oogenesis, egg size |
| c57014_g1_i1 | c48071_g1_i1 | TR37332\|c1_g1_i1 | Calreticulin | 405 | Osmotic stress |
| c53549_g1_i1 | c68409_g1_i1 | TR49044\|c0_g1_i1 | Carbonic Anhydrase | 330 | Ion exchange |
| c58343_g5_i3 | c61294_g1_i1 | TR48638\|c0_g1_i1 | Claudin | 783 | Cellular junction |
| c51607_g1_i1 | c58208_g2_i1 | TR37695\|c0_g1_i1 | Crustacean cardiovascular peptide | 142 | Body fluid |
| c51011_g1_i2 | c66214_g1_i3 | TR50088\|c0_g1_i2 | Crustacean hyperglycemic hormone | 135 | Hemolymph |
| c59199_g1_i1 | c61474_g1_i1 | TR50228\|c0_g1_i3 | Cullin | 829 | Larval development |
| c53414_g1_i1 | c68599_g1_i1 | TR35182\|c0_g1_i2 | Diuretic Hormone | 141 | Water balance |
| c55938_g2_i1 | c68728_g1_i1 | TR48241\|c0_g3_i3 | Heat shock proteins | 685 | Stress tolerance |
| c54868_g1_i1 | c68692_g1_i1 | TR42311\|c1_g1_i3 | H^+^/Cl^-^ exchanger | 386 | Ion channel regulate |
| c59162_g1_i1 | c72724_g1_i2 | TR52560\|c0_g1_i2 | Integrin | 1743 | Cell volume, junction |
| c43728_g2_i1 | c65834_g1_i1 | TR30286\|c0_g1_i2 | ILF2 | 400 | Osmotic stress |
| c44642_g2_i2 | c67441_g2_i1 | TR32838\|c0_g1_i1 | K^+^Cl^-^ symporter | 74 | Ion transport |
| c58691_g1_i3 | c66529_g4_i1 | TR49977\|c0_g1_i1 | Leukocyte ARL | 121 | Oogenesis, egg size |
| c54380_g1_i1 | c70656_g6_i4 | TR47424\|c0_g1_i1 | P38 MAP Kinase | 99 | Osmotic signaling |
| c57613_g2_i2 | c9666_g1_i1 | TR92581\|c0_g1_i1 | Mastermind | 375 | Embryogenesis |
| c52803_g1_i1 | c66356_g1_i1 | TR36397\|c0_g1_i2 | Merlin | 176 | Larval development |
| c60002_g1_i1 | c71277_g2_i2 | TR46215\|c0_g1_i1 | Midline | 900 | Egg size, development |
| c32078_g2_i1 | c65924_g1_i1 | TR44866\|c0_g1_i2 | Mitochondrial carrier protein | 157 | Osmotic signaling |
| c52160_g1_i1 | c60502_g1_i1 | TR49790\|c0_g1_i1 | Mothers against DPP | 438 | Development, egg size |
| c52854_g1_i1 | c65033_g1_i1 | TR18056\|c0_g1_i1 | Mg^+2^ Transporter | 326 | Ion transport |
| c60147_g2_i1 | c65702_g1_i4 | TR30763\|c1_g1_i1 | Na^+^/K^+^ ATPase | 1036 | Ion exchange, balance |
| c60020_g1_i4 | c64395_g3_i2 | TR32397\|c0_g1_i2 | Na^+^/HCO_3_^-^ transporter | 100 | Ion transport, exchange |
| c59059_g1_i2 | c65072_g1_i1 | TR43569\|c0_g1_i2 | Na^+^/Ca^+2^ exchanger | 911 | Ion transport & balance |
| c59709_g1_i3 | c69641_g1_i1 | TR30412\|c0_g2_i1 | Na^+^/K^+^/2Cl^-^ Co-transporter | 1066 | Ion transport, exchange |
| c59083_g1_i4 | c69986_g1_i4 | TR45226\|c1_g1_i1 | Na^+^/H^+^ exchanger | 679 | Ionic balance |
| c46998_g1_i1 | c70269_g1_i6 | TR109097\|c0_g1_i1 | Na^+^/K^+^/Ca^+2^ exchanger | 68 | Ion transport |
| c51768_g1_i1 | c65478_g1_i1 | TR43451\|c0_g1_i1 | Na^+^ transporter | 552 | Ion transporter |
| c58346_g1_i2 | c67441_g2_i1 | TR49704\|c0_g1_i2 | Potassium Channel | 617 | Ion channel activity |
| c57886_g2_i1 | c67421_g1_i1 | TR45473\|c1_g1_i1 | Selenophosphate | 326 | Osmotic stress |
| c59942_g2_i6 | c70008_g3_i3 | TR49996\|c1_g1_i1 | Serpin | 413 | Larval development |
| c57275_g2_i2 | c63240_g1_i1 | TR17071\|c1_g1_i1 | V-type (H^+^) ATPase | 836 | Ion exchange, balance |
| c58700_g3_i2 | c71302_g1_i1 | TR52105\|c0_g1_i1 | Plekstrin homology domain protein | 150 | Egg membrane |
| c38754_g1_i1 | c70855_g1_i1 | TR47580\|c0_g2_i2 | Vitelline membrane protein | 279 | Oogenesis |
| c59247_g1_i2 | c71762_g1_i1 | TR50263\|c0_g1_i1 | Vitellogenin | 198 | Egg membrane & size |
| c9085_g1_i1 | c65752_g1_i1 | TR51629\|c1_g1_i1 | Novel (UNT1) | 91 | Unknown |
| c90672_g1_i1 | c61215_g1_i1 | TR46926\|c0_g1_i5 | Novel (UNT2) | 78 | Unknown |
| c58543_g5_i1 | c60915_g1_i3 | TR52778\|c1_g5_i1 | Novel (UNT3) | 63 | Unknown |
| c48533_g3_i1 | c57756_g1_i1 | TR11884\|c0_g1_i1 | Novel (UNT4) | 57 | Unknown |
| c54412_g1_i1 | c48106_g1_i1 | TR54728\|c0_g1_i1 | Novel (UNT5) | 46 | Unknown |
| c54450_g2_i1 | c64997_g1_i2 | TR30292\|c0_g1_i1 | Novel (UNT6) | 82 | Unknown |
| c77084_g1_i1 | c70265_g1_i2 | TR62084\|c0_g1_i1 | Novel (UNT7) | 71 | Unknown |
| c39049_g1_i1 | c21621_g1_i1 | TR46146\|c0_g2_i1 | Novel (UNT8) | 84 | Unknown |
| c38473_g1_i1 | c65869_g10_i2 | TR63834\|c0_g1_i1 | Novel (UNT9) | 43 | Unknown |
| c90522_g1_i1 | c24013_g1_i1 | TR34269\|c0_g1_i1 | Novel (UNT10) | 68 | Unknown |
| c57107_g2_i1 | c64105_g1_i1 | TR47015\|c3_g17_i1 | Novel (UNT11) | 104 | Unknown |
| c55193_g1_i2 | c68418_g1_i1 | TR18733\|c0_g1_i1 | Novel (UNT12) | 75 | Unknown |
| c25337_g1_i1 | c63082_g1_i1 | TR506\|c0_g1_i1 | Novel (UNT13) | 81 | Unknown |
| c17904_g1_i1 | c60476_g1_i2 | TR707\|c0_g1_i1 | Novel (UNT14) | 66 | Unknown |
| c13547_g1_i1 | c70991_g3_i1 | TR11591\|c0_g1_i1 | Novel (UNT15) | 54 | Unknown |
| c22128_g1_i1 | c68397_g3_i1 | TR34694\|c1_g4_i1 | Novel (UNT16) | 73 | Unknown |

**Table S4** Differential gene expression matrix of the 26 pre-identified osmoregulatory genes (out of 43) and 16 novel transcripts (with a significant salinity effect only) for the three *Macrobrachium* species. For each gene, table represents the log_2_ fold-change (logFC) between 0‰ and 15‰ salinities, and raw P values (P<0.05 has been presented). Positive logFC values indicate higher expression in 15‰ relative to 0‰.

| **Candidate Genes** | ***M*. *australiense*** | | ***M*. *tolmerum*** | | ***M*. *novaehollandiae*** | |
| --- | --- | --- | --- | --- | --- | --- |
|  | logFC | P-value | logFC | P-value | logFC | P-value |
| Alkaline Phosphatase | -11.4 | 0.000 | -8.3 | 0.000 | -3.7 | 0.002 |
| Aquaporin | -4.6 | 0.001 | -2.9 | 0.003 | 7.9 | 0.000 |
| Arginine Kinase | -5.8 | 0.002 | -4.9 | 0.000 | -1.8 | 0.004 |
| ABC Family | -4.3 | 0.000 | -4.1 | 0.001 | 2.2 | 0.000 |
| Ca^+2^-ATPase | -3.6 | 0.003 | -4.1 | 0.000 | -1.6 | 0.008 |
| Calreticulin | 3.9 | 0.000 | 3.8 | 0.002 | 12.2 | 0.000 |
| Carbonic Anhydrase | -6.8 | 0.000 | -3.7 | 0.001 | -1.7 | 0.005 |
| Claudin | 5.7 | 0.000 | 5.9 | 0.000 | 17.4 | 0.000 |
| Crustacean cardiovascular peptide | -10.6 | 0.000 | -9.6 | 0.000 | -2.1 | 0.003 |
| Crustacean hyperglycemic hormone | -12.5 | 0.000 | -11.8 | 0.000 | 8.9 | 0.000 |
| Diuretic Hormone | 4.8 | 0.000 | 5.2 | 0.000 | 13.1 | 0.000 |
| Heat shock proteins | -3.4 | 0.002 | -3.6 | 0.002 | 7.7 | 0.000 |
| H^+^/Cl^-^ exchanger | -2.7 | 0.007 | -2.3 | 0.008 | -1.6 | 0.006 |
| Integrin | 6.2 | 0.000 | 6.8 | 0.000 | 13.8 | 0.000 |
| Interleukin Factor 2 (ILF2) | -10.6 | 0.000 | -8.2 | 0.000 | 12.8 | 0.000 |
| P38 MAP Kinase | -9.6 | 0.000 | -10.2 | 0.000 | 15.3 | 0.000 |
| Mitochondrial carrier protein | -6.7 | 0.000 | -4.5 | 0.007 | 3.9 | 0.009 |
| Na^+^/K^+^ ATPase | -12.6 | 0.000 | -11.2 | 0.000 | 10.6 | 0.000 |
| Na^+^/HCO_3_^-^ transporter | -3.8 | 0.004 | -3.9 | 0.004 | -2.4 | 0.007 |
| Na^+^/Ca^+2^ exchanger | -2.1 | 0.009 | -1.9 | 0.01 | -1.2 | 0.04 |
| Na^+^/K^+^/2Cl^-^ Co-transporter | -14.7 | 0.000 | -12.6 | 0.000 | -7.9 | 0.000 |
| Na^+^/H^+^ exchanger | 6.8 | 0.000 | 7.3 | 0.000 | 11.2 | 0.000 |
| Na^+^/K^+^/Ca^+2^ exchanger | -3.1 | 0.003 | -3.8 | 0.01 | -2.1 | 0.03 |
| Na^+^ transporter | -2.7 | 0.02 | -2.2 | 0.02 | -2.4 | 0.03 |
| Selenophosphate | -6.9 | 0.000 | -7.3 | 0.000 | 8.6 | 0.000 |
| V-type (H^+^) ATPase | 5.9 | 0.000 | 7.2 | 0.000 | 14.6 | 0.000 |
| UNT1 | -4.8 | 0.001 | -5.3 | 0.000 | -2.8 | 0.02 |
| UNT2 | -7.9 | 0.000 | -6.6 | 0.000 | -3.2 | 0.01 |
| UNT3 | -8.5 | 0.000 | -8.8 | 0.000 | -3.8 | 0.008 |
| UNT4 | -9.6 | 0.000 | -5.7 | 0.000 | -2.9 | 0.009 |
| UNT5 | -3.6 | 0.004 | -4.2 | 0.001 | -4.6 | 0.002 |
| UNT6 | -4.8 | 0.001 | -3.5 | 0.006 | 5.7 | 0.000 |
| UNT7 | -6.5 | 0.000 | -4.4 | 0.02 | 4.3 | 0.002 |
| UNT8 | -2.6 | 0.004 | -2.3 | 0.04 | 3.8 | 0.004 |
| UNT9 | -4.4 | 0.002 | -5.4 | 0.000 | 2.7 | 0.01 |
| UNT10 | -3.8 | 0.005 | -2.9 | 0.008 | 4.4 | 0.000 |
| UNT11 | 3.7 | 0.001 | 4.3 | 0.000 | 5.6 | 0.000 |
| UNT12 | 2.7 | 0.02 | 2.9 | 0.01 | 4.9 | 0.001 |
| UNT13 | 4.9 | 0.000 | 4.1 | 0.003 | 4.2 | 0.002 |
| UNT14 | 8.1 | 0.000 | 9.2 | 0.000 | 3.4 | 0.02 |
| UNT15 | 5.4 | 0.001 | 5.1 | 0.002 | 2.9 | 0.04 |
| UNT16 | 3.8 | 0.002 | 4.3 | 0001 | 3.3 | 0.000 |

**Table S5** List of positively selected genes for MA vs MT and MA vs MN comparisons. MA = *M*. *australiense*, MT = *M*. *tolmerum*, MN = *M*. *novaehollandiae*

| **Species Pair** | **Gene (Functional Roles)** | **d_N_** | **d_S_** | **d_N_/d_S_** | **f_N_** | **P-value** |
| --- | --- | --- | --- | --- | --- | --- |
| MA vs MT  (ALD vs ELD) | Merlin (larval development) | 0.0653 | 0.0334 | 1.9551 | 0.6561 | 0.003 |
|  | Mastermind (larval development) | 0.0713 | 0.0406 | 1.7561 | 0.6121 | 0.005 |
|  | Midline (multiple larval developmental role) | 0.0217 | 0.0183 | 1.1858 | 0.5129 | 0.004 |
|  | Selanophosphate (osmotic stress tolerance) | 0.0122 | 0.0117 | 1.0427 | 0.5170 | 0.000 |
|  | Calreticulin (osmotic stress response) | 0.0209 | 0.0202 | 1.0346 | 0.5017 | 0.000 |
|  | P38 MAP kinase (osmotic signal transduction) | 0.0124 | 0.0116 | 1.0689 | 0.5142 | 0.006 |
|  | Interleukin (signaling for osmotic stress) | 0.0218 | 0.0184 | 1.1982 | 0.5415 | 0.002 |
|  | Claudin (maintain cellular junction) | 0.0357 | 0.0248 | 1.4395 | 0.5886 | 0.001 |
|  | Integrin (maintain cell volume & junction) | 0.0408 | 0.0309 | 1.3204 | 0.5671 | 0.001 |
|  | Aquaporin (water channel regulation) | 0.0546 | 0.0398 | 1.3718 | 0.5692 | 0.003 |
|  | Diuretic hormone (water balance) | 0.0648 | 0.0491 | 1.3198 | 0.5689 | 0.001 |
|  | Hyperglycemic hormone (regulate body fluid) | 0.0736 | 0.0486 | 1.5144 | 0.6023 | 0.002 |
|  | Vitellogenin (controlling egg size) | 0.0815 | 0.0478 | 1.7050 | 0.6323 | 0.000 |
|  | Vitelline (controlling egg size) | 0.0402 | 0.0393 | 1.0229 | 0.5043 | 0.000 |
|  | Serpin (controlling egg size & development) | 0.0235 | 0.0219 | 1.0730 | 0.5209 | 0.007 |
|  | Cullin (controlling egg size) | 0.0172 | 0.0137 | 1.2554 | 0.5614 | 0.000 |
|  | Plekstrin (controlling egg size) | 0.0216 | 0.0174 | 1.2414 | 0.5498 | 0.006 |
|  | Growth-arrest-specific protein (growth inhibition) | 0.0208 | 0.0174 | 1.1954 | 0.5213 | 0.001 |
|  | Heparan sulphate 6 (growth) | 0.0254 | 0.0201 | 1.2637 | 0.5086 | 0.000 |
|  | Alpha amylase (growth) | 0.0702 | 0.0413 | 1.6998 | 0.6023 | 0.000 |
|  | Pantothanate flavoprotein (metabolic activity) | 0.0659 | 0.0328 | 2.0091 | 0.7081 | 0.003 |
|  | Glutatheone synthetase (Feeding behavior) | 0.0285 | 0.0179 | 1.5922 | 0.6358 | 0.000 |
|  | RAP guanine factor 4 (energy budgeting) | 0.0407 | 0.0291 | 1.3986 | 0.5157 | 0.001 |
|  | Syndecan isoform 2 (energy homeostasis) | 0.0116 | 0.0113 | 1.0265 | 0.5076 | 0.009 |
|  | TKT protein (energy production) | 0.0211 | 0.0184 | 1.1467 | 0.5108 | 0.002 |
|  | Ubiquitin C (metabolic process) | 0.0362 | 0.0298 | 1.2148 | 0.5134 | 0.001 |
| MA vs MN  (ALD vs ELD) | Selanophosphate | 0.0284 | 0.0141 | 2.0142 | 0.6682 | 0.000 |
|  | Calreticulin | 0.0294 | 0.0207 | 1.4202 | 0.5868 | 0.001 |
|  | P38 MAP kinase | 0.0186 | 0.0119 | 1.5630 | 0.6098 | 0.002 |
|  | Interleukin | 0.0274 | 0.0185 | 1.4811 | 0.5969 | 0.000 |
|  | Claudin | 0.0421 | 0.0263 | 1.6008 | 0.6155 | 0.001 |
|  | Integrin | 0.0497 | 0.0307 | 1.6189 | 0.6182 | 0.000 |
|  | Aquaporin | 0.0631 | 0.0395 | 1.5975 | 0.6150 | 0.001 |
|  | Diuretic hormone | 0.0732 | 0.0492 | 1.4878 | 0.5980 | 0.001 |
|  | Hyperglycemic hormone | 0.0684 | 0.0345 | 1.9826 | 0.6647 | 0.004 |
|  | Vitelline | 0.0524 | 0.0386 | 1.3575 | 0.5758 | 0.000 |
|  | Vitellogenin | 0.0874 | 0.0451 | 1.9379 | 0.6596 | 0.001 |
|  | Serpin | 0.0253 | 0.0197 | 1.2843 | 0.5622 | 0.005 |
|  | Cullin | 0.0216 | 0.0141 | 1.5319 | 0.6050 | 0.000 |
|  | Plekstrin | 0.0319 | 0.0171 | 1.8655 | 0.6510 | 0.002 |
|  | Midline | 0.0286 | 0.0189 | 1.5132 | 0.6021 | 0.000 |
|  | Merlin | 0.0716 | 0.0329 | 2.1763 | 0.6852 | 0.000 |
|  | Mastermind | 0.0772 | 0.0381 | 2.0263 | 0.6695 | 0.004 |
|  | Growth-arrest-specific protein | 0.0205 | 0.0170 | 1.2059 | 0.5421 | 0.000 |
|  | Heparan sulphate 6 | 0.0259 | 0.0203 | 1.2759 | 0.5182 | 0.000 |
|  | Alpha amylase | 0.0710 | 0.0415 | 1.7108 | 0.6152 | 0.000 |
|  | Pantothanate flavoprotein | 0.0671 | 0.0332 | 2.0211 | 0.7108 | 0.002 |
|  | Glutatheone synthetase | 0.0289 | 0.0180 | 1.6056 | 0.6471 | 0.000 |
|  | RAP guanine factor 4 | 0.0418 | 0.0289 | 1.4464 | 0.5251 | 0.000 |
|  | Syndecan isoform 2 | 0.0123 | 0.0114 | 1.0789 | 0.5009 | 0.007 |
|  | TKT protein | 0.0216 | 0.0182 | 1.1868 | 0.5129 | 0.001 |
|  | Ubiquitin C | 0.0369 | 0.0296 | 1.2466 | 0.5217 | 0.000 |
